# Interferon therapy and its association with depressive disorders – A review

**DOI:** 10.3389/fimmu.2023.1048592

**Published:** 2023-02-22

**Authors:** Jing Yung Lai, Jian Xiang Ho, Audrey Siew Foong Kow, Gengfan Liang, Chau Ling Tham, Yu-Cheng Ho, Ming Tatt Lee

**Affiliations:** ^1^ Faculty of Pharmaceutical Sciences, UCSI University, Kuala Lumpur, Malaysia; ^2^ Department of Biomedical Sciences, Faculty of Medicine and Health Sciences, Universiti Putra Malaysia, Serdang, Selangor, Malaysia; ^3^ School of Medicine, College of Medicine, I-Shou University, Kaohsiung City, Taiwan

**Keywords:** depression, neuroinflamamation, interferons, adverse (side) effects, hippocampus, HPA axis

## Abstract

Interferons (IFNs) are important in controlling the innate immune response to viral infections. Besides that, studies have found that IFNs also have antimicrobial, antiproliferative/antitumor and immunomodulatory effects. IFNs are divided into Type I, II and III. Type I IFNs, in particular IFN-α, is an approved treatment for hepatitis C. However, patients developed neuropsychological disorders during treatment. IFN-α induces proinflammatory cytokines, indoleamine 2,3-dioxygenase (IDO), oxidative and nitrative stress that intensifies the body’s inflammatory response in the treatment of chronic inflammatory disease. The severity of the immune response is related to behavioral changes in both animal models and humans. Reactive oxygen species (ROS) is important for synaptic plasticity and long-term potentiation (LTP) in the hippocampus. However, excess ROS will generate highly reactive free radicals which may lead to neuronal damage and neurodegeneration. The limbic system regulates memory and emotional response, damage of neurons in this region is correlated with mood disorders. Due to the drawbacks of the treatment, often patients will not complete the treatment sessions, and this affects their recovery process. However, with proper management, this could be avoided. This review briefly describes the different types of IFNs and its pharmacological and clinical usages and a focus on IFN-α and its implications on depression.

## 1 Introduction

Neuroinflammation in the central nervous system has been suggested to play a role in the pathophysiology of depression and anxiety (1–3). Interferons (IFN), a family of proinflammatory cytokines, are used in the treatment of a variety of autoimmune (e.g. multiple sclerosis), viral (e.g. chronic hepatitis B and C), and malignant (e.g. malignant melanoma, hairy cell leukemia) disorders. However, mood disorders such as depression and anxiety are notably the serious adverse effects that are associated with IFN therapy (4). Chronic administration of interferons impacts the neuronal function of hippocampal tissue, in which the hippocampal neurogenesis significantly declined in patients treated with interferon-α (5). It is believed that hyperfunctional hypothalamic-pituitary-adrenal (HPA) axis, at least in part, contributes to the depressive and anxiety emotions-associated with interferon treatment (6, 7). Suicidal thought and actual suicidal cases were reported to be associated with interferon administration in patients (8). In this review, evidences of IFN-α-induced depression were presented together with its possible mechanisms; with a brief introduction on the different types of interferons and their use as pharmacotherapies.

## 2 Classifications of interferon

### 2.1 Type I interferons

Type I IFNs comprised of multiple subtypes of IFN-α, a single IFN-β and the less characterized IFN-δ, -ϵ, -κ, -ζ, -τ, and -ω (9). Both type I and type III IFNs activate the same antiviral pathways but through different receptors (10). Type I IFNs will bind to the heterodimeric receptor complex IFNAR1 and IFNAR2 and activate the receptor-associated tyrosine kinases tyrosine kinase 2 (TYK2) and Janus kinase (JAK) 1 (9). IFNAR1 will bind specifically to TYK2 while IFNAR2 will bind to JAK1 of the JAK family (11). This will then phosphorylate STAT1 and STAT2 forming a trimeric complex known as IFN-stimulated gene factor 3 (ISGF3) which will enter the nucleus and bind to IFN-stimulated response elements (ISREs) promoting the transcription of hundreds of IFN-stimulated genes (9). ISGF3 is a complex of phosphorylated STAT1, STAT2 and unphosphorylated interferon regulatory factor 9 (IRF-9). All seven STAT family members can be activated by type I IFNs in different cell types. This causes the formation of many hetero- and homodimer pairs and complexes with other transcription factors (11). As for Type III IFNs, they will bind onto IFNLR1 and IL-10R2. Upon binding, the TYK2 will be activated, ISGF3 formed and ISG expressed just like in type I IFNs activation pathway (9).

Type I IFN are rapidly induced and act against viral infection at any stage of the virus life cycle. The antiviral activity occurs in an autocrine and paracrine manner in surrounding cells by inducing the transcription of genes involved in apoptosis, anti-growth, and innate and adaptive immune cell activation (12). Type I IFN induce several anti-viral genes such as the protein kinase R (PKR), adenosine deaminase acting on RNA (ADAR), 2’,5’-oligoadenylate synthetase (OAS), cellular ribonuclease (RNase) L and Mx proteins among others. In a murine model for human cytomegalovirus infection – murine cytomegalovirus (MCMV) clearance requires the activation of natural killer (NK) cells to detect MCMV-infected cells by Ly49H (NK receptor) whereby NK cells are activated by type I IFN through inducing cytokines (12). Apart from inducing proteins with direct antiviral effects, type I IFNs also regulate the innate and adaptive immune systems. For instance, the function of NK cells is regulated by type I IFNs whereby they induce the production of interleukin-15 (IL-15) promoting the survival and proliferation of NK cells. Natural cytotoxicity triggering receptor 1 (NCR1) activates NK cells and promotes cellular apoptosis in viral-infected, cancer cells and inappropriately activated T cells. IFN-α could inhibit NCR1 expression, decreasing NK cells level and protect CD8^+^ and CD4^+^ T cells from destruction by NK cells (13). IFN-α can directly activate NK cells to produce IFN-γ cytokine or indirectly control NK cell function *via* STAT1 and 4 gene transcriptions. Type I IFNs can activate dendritic cells (DC) too, by promoting the differentiation of DC from monocyte precursors and are powerful activators of DC function *in vitro* and *in vivo*. *In vivo* stimulation of DC with type I IFN showed potent humoral and cellular immune responses to protein antigens. DC can produce type I IFN under steady-state conditions and upon viral stimulation. The produced type I IFN can act in both autocrine and paracrine manner to stimulate DC (14). Type I IFNs too were found to possibly induce differentiation of pDC subsets into mature antigen presenting cells (APCs) (12). Type I IFN also play a role in activating naive CD8^+^ T cells, survival of activated CD4^+^ and CD8^+^ cells and development and proliferation of B cells (12). Endogenous type I IFNs are also able to suppress immune activity. In experimental autoimmune encephalomyelitis (EAE), high levels of IFN-β and other type I IFNs are noted at sites of inflammation. In addition, the expression of IFN-β in the central nervous system inhibits EAE by reducing expression of chemokines, production of proinflammatory cytokines and the antigen presentation ability of myeloid cells (15).

On the flip side, type I IFNs may be detrimental to the host. In the case of intracellular bacterial pathogen infection *Listeria monocytogenes* (*L. monocytogenes*), type I IFNs will sensitize macrophages and lymphocytes to cell death. Residing in the macrophages, intestinal epithelial cells and hepatocytes, *L. monocytogenes* causes the production of type I IFN which sensitize infected splenocytes to *L. monocytogenes*-induced apoptosis. As a result of splenocytes apoptosis, IFN-induction of proapoptotic gene programs – PKR, death-receptor ligand TRAIL and Daxx will also be activated (12). Similarly, during *Mycobacterium tuberculosis* (Mtb) infection, expressions of both IFN-α and IFN-β are induced and high levels of these IFN have shown to kill infected mice as they failed to elicit proper Th1 responses as summarized by Perry et al. (12). Other detrimental effects of type I IFNs include enhancing the pathogenesis of autoimmune diseases such as systemic lupus erythematosus, type I diabetes (12), Sjögren’s syndrome, neuromyelitis optica, rheumatoid arthritis and psoriasis (15).

#### 2.1.1 Pharmacotherapy applications of IFN-α

IFN-α2 treatment for hepatitis C (HCV) has been the main therapy of choice to prevent the infection from further deterioration (16). The introduction of pegylated IFN (PEG-IFN) for chronic HCV infection has seen tremendous positive response. There are two commercially available PEG-IFN that are widely used clinically. They derived from IFN-α2a and IFN-α2b. With PEG-IFN and ribavirin therapy, sustained virological response (SVR) rates have risen to between 54% and 63% compared to less than 15% of conventional IFN monotherapy (17). The SVR rates is the absence of detectable HCV RNA in a patient’s blood 6 months after completing the antiviral treatment (18). Rapid decline of HCV RNA levels in IFN-α-treated patients was suggested to be caused by the inhibition of viral production by IFN as demonstrated by Rong and Perelson’s viral dynamic model. As the viral load drops, the rate of *de novo* infection also drops, and the level of infected cells could not be maintained. This in turn led to a slow net loss of infected cells (18). The use of IFN-α therapy on hepatitis B virus infection (HBV) is also approved and it worked by modulating the immune system. A weak direct anti-viral effect was also noted. Even though there was not much difference between the efficacy of conventional IFN-α and PEG-IFN-α2a therapy, the HBeAg seroconversion rate which correlates with SVR was above 30% (16).

IFN-α has well known effect on tumor size reduction and halt progression of tumors especially during G1 cancer phase. IFN-α activates cyclin-dependent kinase inhibitor (CDKN)1A gene transcription to either block G1 cancer phase or prolong time for cell undergoing mitosis. IFN-α induced STAT1 gene transcription increases tumor necrosis factor-alpha (TNF-α) proinflammatory cytokine accompany with disruption of mitochondrial integrity and release of Cytochrome C to initiate caspase-8 pathway in promoting malignant cell apoptosis (19). IFN-α has indirect effects on cancer cells such as angiogenesis inhibition and immune response. IFN-α reduces vascular endothelial growth factor (VEGF) to prevent migration and proliferation of endothelial cells leading to ischemic necrosis on tumor micro-vessels (20).

Early treatment with IFN-α injections was found to reduce the attack length and/or severity in patients with colchicine-resistant familial Mediterranean fever (FMF). FMF is an autoinflammatory disease. Patients suffer peritoneal, pleural, and synovial inflammation attacks and gradual development of amyloidosis. Colchicine is the only effective therapy to prevent FMF attacks. However, there are about 10% of patients who do not respond to colchicine. IFN-α was first noticed as a possible drug for prophylaxis of FMF by Tankurt et al. (21) in a patient with uncontrolled FMF attacks on top of hepatitis C infection and was receiving IFN-α treatment. Subsequently Tunca et al. (22) also saw that in 7 FMF patients treated with IFN-α, 18 out of 21 attacks were stopped within 3 hours (mean). It also attenuated pain intensity in these patients. However, Tunca et al. in 2004 (23) also reported unfavorable response of IFN-α on FMF and this could be due to the late administration of the drug. Following that 2 more groups published supporting results that favored the use of IFN-α as treatment for FMF (24).

#### 2.1.2 Pharmacotherapy applications of IFN-β

IFN-β, is a polypeptide that is produced by fibroblasts and has antiviral and antiproliferative activities (25). It has been used to treat multiple sclerosis (MS) due to its immunomodulatory property (26). MS is a neurodegenerative disease that is progressive, and it is associated with demyelination of nerves (26). IFN-β reduces autoimmune destruction of myelin sheath in MS by retaining the integrity of the blood-brain-barrier (BBB). IFN-β reduces antigen presentation and T-cell proliferation (27). It also changes the expression of cytokine (suppressed TH_1_ proinflammatory cytokines; promote TH_2_ anti-inflammatory cytokine) and matrix metalloproteinase (MMP) expression restoring suppressor function (25). IFN-β therapy has two different formulations that mimic IFN-β action exogenously. IFN-β1a is naturally occurring amino acids extracted from Chinese Hamster Ovary cells which is glycosylated. IFN-β1b is produced by prokaryotic gene expression by deleting N-terminal methionine and substituting serine with cysteine residue at position 17 which is not glycosylated. IFN-β1a is usually preferred over IFN-β1b because it has a higher potency in antiviral activity and glycosylation reduced immunogenicity. Both have different routes of administration - IFN-β1a is given in intramuscular injection due to immunogenicity, meanwhile IFN-β1b is administered subcutaneously due to higher low immunogenicity (25). When administered together with natalizumab, a recombinant monoclonal antibody to an integrin, the rate of MS progression was greatly reduced. A reduction by 50% in the rate of MS relapses was achievable in IFN-β and natalizumab combined treatment compared to that observed in IFN-β only treatment (26). IFN-β encourages maturation of dendritic cells only when it is insufficient but interrupts maturation process of dendritic cell in overload situation. Further investigation on the effects of IFN-β treatment of MS found that IFN-β also prevented the migration of dendritic cell from inflammatory regions to draining lymph nodes for antigen presentation and activation of naive T cells. This was demonstrated through the suppression of C-C chemokine receptor-7 (CCR7) and matrix metalloproteinase (MMP)-9 through STAT1 signaling pathway (28). Early IFN-β-1a subcutaneous therapy given three times weekly was found beneficial in patients with relapsing-remitting MS over an 8-year period. Patients given the higher dose of 44 μg IFN-β-1a recorded lower Expanded Disability Status Scale progression, relapse rate and T2 burden of disease up to 8 years, while patients treated 2 years later showed otherwise (29).

IFN-β therapy has been used as antiviral agents against human immunodeficiency virus- 1 (HIV-1) encephalitis, Hepatitis B and C, herpes, zoster, herpes simplex virus (HSV) and cytomegalovirus infection (27). It is also used to treat rheumatoid arthritis (RA) and this was attributed to its anti-inflammatory property in reducing key proinflammatory cytokines of RA – interleukin-6 (IL-6) and TNF-α, matrix metalloproteinases (MMPs) and prostaglandin E2. IFN-β’s antiangiogenic property also enhances the therapeutic effect in RA (30). IFN-β could also be used as a co-therapy agent with other chemotherapeutics in chronic myelogenous leukemia, condyloma acuminate and melanomas attributed to its antitumor activity as shown in *in vitro* studies (31). IFN-β may be used to treat Ebola virus patients as it was shown effective in inhibiting Ebola virus *in vitro* by McCarthy et al. (32) and in monkeys by Smith et al. (33). McCarthy et al. (32) found that both IFN-α and -β were able to inhibit Ebola virus replication 24 hour (h) post-infection with IC_50_ of 0.038 μM and 0.016 μM respectively. The inhibition was augmented when IFN-β was administered together with lamivudine (97.3% inhibition); and the triple combination of IFN-β, lamivudine and zidovudine also showed high inhibition of the virus at 95.8% (32). Early postexposure IFN-β treatment increased survival time of Ebola virus- and Marburg virus-infected rhesus macaques. Though the treatment did not prevent mortality, Smith et al. (33) suggested that IFN-β therapy could likely be used as an adjunct to other agents and this will certainly require further studies.

IFN-β therapy is effective in both TH_1_-driven and TH_17_-driven autoimmunity diseases such as EAE and relapsing remitting multiple sclerosis (RRMS) as IFN-β blocks TH_1_ associated pathologies *via* the inhibition of inflammatory interferon-gamma (IFN-γ) and interleukin-12 (IL-12) (15). IFN-β also inhibits TH_17_ cells differentiation. However, there are a subset of RRMS patients who do not respond to IFN-β therapy, whereby the therapy induced gene expression, exacerbating RRMS. Increased levels of both TH_17_ and type I IFN were seen in the blood of these non-responders before the start of treatment, suggesting that the pathology of RRMS was initiated by TH_17_ cells. In these patients, IFN-β will not work as endogenous IFN-β expression is already high (15). Therefore, IFN-β therapy should be administered with caution. A compilation of the clinical trials of IFN-α and -β and their results were presented in **Tables 1**, **2**.

**Table 1:** Clinical trials of IFN-α and their results.

Identifier Number (Study Date)	Clinical Trial Title	Treatment Arms			Study Results	References
NCT02761629 (April 2005 – May 2008)	Randomized, Multi-Center, Phase IV, Comparative Study to Assess the Efficacy of Combined Peg-Interferon Alpha-2a (40 kD) with Ribavirin Combined Therapy for 48 or 72 weeks of Treatment and 24 weeks of Follow-Up in Patients with Chronic Hepatitis C, Genotype 1, Co-Infected with Human Immunodeficiency Virus	Participants received 180 micrograms (mcg) of Peg-IFN-Alpha-2A (once weekly, subcutaneous injection) and ribavirin [1000 milligrams (mg) or 1200 mg] for 48 weeks	Participants received 180 micrograms (mcg) of Peg-IFN-Alpha-2A (once weekly, subcutaneous injection) and ribavirin [1000 milligrams (mg) or 1200 mg] for 72 weeks		1. 36.5% of participants experienced Sustained Virologic Response (SVR) with 72 weeks of treatment. 2. 56.9% of subjects have undetectable HCV RNA with 72 weeks of treatment.	(34)
NCT00491244 (June 2007 - September 2013)	Pegylated Interferon Alfa-2a Plus Low Dose Ribavirin Versus Pegylated Interferon Alfa-2a Alone for Treatment-naive Hemodialysis Patients with Chronic Hepatitis C	Participants received pegylated interferon alfa-2a 135 ug/week plus ribavirin 200 mg/day for 24 to 48 weeks	Participants received pegylated interferon alfa-2a 135 ug/week for 24 to 48 weeks		Significant difference (p < 0.001) in SVR between participants receiving peginterferon and ribavirin compared to peginterferon only	(35)
NCT01095835 (February 2005 - January 2010)	A Multicenter, Randomized, Controlled Study Comparing the Efficacy and Safety of 48 weeks of 40 kD Branched Pegylated Interferon Alfa-2a (PEG-IFN, RO 25-8310) Versus 96 Weeks of PEG-IFN, Alone or in Combination with 100 mg Lamivudine for 48 Weeks in Patients with HBeAg-Negative Chronic Hepatitis B	Participants received 180 mcg PEG-IFN (once weekly, subcutaneously) for 48 weeks	Participants received 180 mcg PEG-IFN (once weekly, subcutaneously) for 48 weeks, followed by 135 mcg PEG-IFN (once weekly subcutaneously) for another 48 weeks	Participants received 180 mcg PEG-IFN (once weekly, subcutaneously) for 48 weeks, followed by 135 mcg PEG-IFN (once weekly subcutaneously) for another 48 weeks and Lamivudine (100 mg, daily, orally) from Week 0 to 48	1. 11.8% of participants achieved combined response at the end of the follow-up period with PEG-IFN alfa-2a SC 180 mcg/week for 48 weeks 2. 25% of participants achieved combined response at the end of the follow-up period with PEG-IFN alfa-2a SC 180 mcg/week for 48 weeks followed by 135 mcg/week from 49 to 96 weeks 3. 20% of participants achieved combined response at the end of the follow-up period with Lamivudine PO 100mg/week and PEG-IFN alfa-2a for 48 weeks followed by PEG-IFN alfa-2a SC 135 mcg/week from 49 to 96 weeks	(36)
NCT02263079 (June 16, 2014 – January 29, 2020)	A Phase IIIb, Randomized, Open-Label Study of Pegylated Interferon Alfa-2A in Combination with Lamivudine or Entecavir Compared with Untreated Control Patients in Children with HBeAg Positive Chronic Hepatitis B in the Immune-Tolerant Phase	Participants received either Entecavir (film-coated tablet/oral solution, once daily, 0.015 mg/kg) or Lamivudine (film-coated tablet/oral, once daily, 3 mg/kg) for 8 weeks followed by peg-IFN-alfa-2A (180 mcg/1.73m^2^ body surface area, BSA) combined with either Entecavir or Lamivudine for 48 weeks	Untreated Control Participants (observed up to 80 weeks)	Participants received Peginterferon Alfa 2A (180 mcg/1.73m^2^ BSA, once weekly, subcutaneously) for 48 weeks	3.8% of participants show eradication of HBsAg after 24 weeks of treatment compared to untreated control participants	(37)
NCT00594880 (January 2008 – May 2011)	Antiviral Activity of Peg-IFN-Alpha-2A in Chronic HIV-1 Infection	Participants received 180 mcg Peg-IFN-Alpha-2A (once weekly, subcutaneous) for 24 weeks; 5 weeks with ART, then 7 weeks without ART and further 12 weeks without ART	Participants received 90 mcg Peg-IFN-Alpha-2A (once weekly, subcutaneous) for 24 weeks; 5 weeks with ART, then 7 weeks without ART and further 12 weeks without ART		1. Pegasys dose of 90 mcg/week has better outcome in suppression of HIV Viral Load than 180 mcg/week. 2. 12 weeks of 90mcg/week Pegasys treatment successfully reduced HIV Viral Load below 400 copies/ml in 50% of participants while 24 weeks of 90mcg/week Pegasys treatment successfully reduced HIV Viral Load below 400 copies/mL in 80% of participants. 3. 30% of participants had HIV Viral Load below 48 copies/ml in 12 weeks of 90mcg/week Pegasys treatment.	(38)
NCT00719264 (November 12, 2008 – April 15, 2013)	A Randomized, Open-Label, Multicenter Phase II Study to Compare Bevacizumab Plus RAD001 Versus Interferon Alfa-2a Plus Bevacizumab for the First-line Treatment of Patients with Metastatic Clear Cell Carcinoma of the Kidney	Participants received RAD001 (Everolimus, oral, 10 mg qd) plus Bevacizumab (10 mg/kg, intravenous, every 2 weeks)	Participants received interferon alfa-2a (3 million international unit, MIU, week 1; 6 MIU, week 2; 9 MIU, week 3 and subsequently, 3 times/week) plus Bevacizumab (10 mg/kg, intravenous, every 2 weeks)		1. 9.3% of participants with Bevacizumab and Everolimus combination shows progression-free survival 2. 10% of participants with Interferon Alfa-2a and Bevacizumab combination shows progression-free survival 3. Combination of anti-cancer drugs with Interferon Alfa-2a have better efficacy in halting progression of tumor.	(39)
NCT00525031 (August 2006 – June 2016)	Randomized Phase II Neoadjuvant Study of Temozolomide Alone or with Pegylated Interferon-alpha 2b in Patients with Resectable American Joint Committee on Cancer (AJCC) Stage IIIB/IIIC or Stage IV (M1a) Metastatic Melanoma	Participants received Temozolomide (TMZ, 150 mg/m^2^ by mouth (once daily for 7 days, followed by 7 days off; alternating weekly) for 8 weeks	Participants received Temozolomide (150 mg/m^2^ by mouth (once daily for 7 days, followed by 7 days off; alternating weekly) for 8 weeks plus pegylated interferon alpha-2b (0.5 mcg/kg, subcutaneous injection, once weekly) for 8 weeks		1. 15.4% of participants show positive clinical response toward tumor cell and 53.8% of participants show progression of tumor growth with Temozolomide (TMZ) PO 150 mg/m2/day for 8 weeks alternately (7 days treatment followed by 7 days off) 2. 29.2% of participants show positive clinical response toward tumor cell and 45.8% of participants show progression of tumor growth in combination of TMZ with PEG-IFN alfa-2b SC 0.5 mcg/kg/week for 8 weeks.	(40)

**Table 2:** Clinical trials of IFN-β and their results.

Identifier Number (Study Date)	Clinical Trial Title	Treatment Arms			Study Results	References
NCT00906399 (June 2009 – October 2013)	A Multicenter, Randomized, Double-Blind, Parallel-Group, Placebo-Controlled Study to Evaluate the Efficacy and Safety of PEGylated Interferon Beta-1a (BIIB017) in Subjects with Relapsing Multiple Sclerosis	Participants received placebo (0.5 mL, subcutaneous, self-administered) every 2 weeks for 48 weeks followed by 125 μg peginterferon beta-1a every 2 or 4 weeks for 48 weeks (subcutaneous, self-administered)	Participants received 125 μg peginterferon beta-1a (subcutaneous, self-administered) every 2 weeks for 96 weeks	Participants received 125 μg peginterferon beta-1a (subcutaneous, self-administered) every 4 weeks for 96 weeks. Participants also received placebo injection 2 weeks after each active injection	25.6% of Annualized Relapse Rate with peginterferon beta-1a SC 125mcg every 2 weeks for 48 weeks compared to 39.7% of Annualized Relapse Rate with placebo.	(41)
NCT01332019 (April 2011 – October 2015)	A Dose-Frequency Blinded, Multicenter, Extension Study to Determine the Long-Term Safety and Efficacy of PEGylated Interferon Beta-1a (BIIB017) in Subjects with Relapsing Multiple Sclerosis	Participants received 125 μg peginterferon beta-1a (subcutaneous) every 4 weeks for at least 2 years to 4 years	Participants received 125 μg peginterferon beta-1a (subcutaneous) every 2 weeks for at least 2 years to 4 years		1. 14.2% of Annualized Relapse Rate with peginterferon beta-1a SC 125mcg every 2 weeks superior to 18.9% of Annualized Relapse Rate with peginterferon beta-1a SC 125mcg every 4 weeks for at least 2 years. 4.75% of participants discontinued treatment due to adverse event. 30.8% of participants experienced high ALT and 20.8% of participants experienced high AST. 60.6% of participants experienced hyperglycemia. 70.8% of participants have elevated plasma protein.	(42)
NCT00883337 (April 2009 – May 2015)	A Multi-center, Randomized, Parallel-group, Rater-blinded Study Comparing the Effectiveness and Safety of Teriflunomide and Interferon Beta-1a in Patients with Relapsing Multiple Sclerosis Plus a Long-Term Extension Period	Participants received 7 mg Teriflunomide (once daily, oral, core treatment period) and 14 mg Teriflunomide (once daily, oral, extended treatment)	Participants received 14 mg Teriflunomide (once daily, oral, core treatment period) and 14 mg Teriflunomide (once daily, oral, extended treatment)	Participants received ascending doses from 8.8 to 44 mcg interferon β-1a (3 times/week, subcutaneous, core treatment period) and 14 mg Teriflunomide (once daily, oral, extended treatment)	1. 48.6% of participants had successful treatment without treatment discontinuation or disease relapse and 41% of Annualized Relapse Rate with Teriflunomide 7 mg OD followed by Teriflunomide 14 mg OD 2. 62.2% of participants had successful treatment without treatment discontinuation or disease relapse and 25.9% of Annualized Relapse Rate with Teriflunomide 14 mg OD. 3. 57.7% of participants had successful treatment without treatment discontinuation or disease relapse and 21.6% of Annualized Relapse Rate with Interferon Beta-1a 3 times a week followed by Teriflunomide 14 mg OD.	(43)
NCT00616434 (May 2008 – March 2010)	A Multicenter, Randomized, Double-Blind, Placebo-Controlled Study to Evaluate the Safety, Tolerability, and Efficacy of Avonex^®^ in Subjects with Moderate to Severe Ulcerative Colitis	Participants received 30 μg interferon beta-1a (twice weekly, intramuscular) for 12 weeks	Participants received placebo (twice weekly, intramuscular) for 12 weeks		1. 53% of participants showed clinical response with Interferon beta-1a IM 30 mcg twice weekly for 12 weeks superior to 44% of participants show clinical response with placebo. 2. 52% of participants experienced adverse event from with Interferon beta-1a treatment.	(44)
NCT00048347 (October 2002 – May 2010)	An Open-Label, Pilot Study of Type I Interferon (AVONEX) Treatment of Ulcerative Colitis	Participants received 30 μg interferon-beta1a every week for 12 weeks			63% of participants with at least 3 points drop in the Short Clinical Colitis Score (SCCAI) with 30 mcg IM weekly for 12 weeks.	(45)

### 2.2 Type II interferon

There is only one single member representing the Type II IFN group which is IFN-γ (9). IFN-γ is secreted by NK cells and T cells i.e., not directly by virus-infected cells. Type II IFN will bind to its respective receptors composed of IFNGR1 and IFNGR2 and signals through JAK1 and JAK2 promoting the phosphorylation and homodimerization of STAT1 leading to the expression of gamma interferon activation site (GAS)-regulated genes as summarized by Kim and Shin (9).

Besides playing a role in both innate and adaptive immune response against pathogens and tumors, IFN-γ, a pleotropic cytokine is also important in maintaining immune homeostasis (46). IFN-γs are able to activate macrophages as seen in many *in vitro* studies in the early 1980s and this phenomenon enhances their ability to kill different types of ingested microorganisms. For instance, exposure of cultured mouse peritoneal cells with *Leishmania tropica* to IFN-γ saw the killing of the organism. Similarly, IFN-γ also demonstrated cytocidal effect on tumor cells and extracellular *Schistosomula*. The anti-microbial activity of IFN-γ was further confirmed with the availability of pure IFN-γ and specific antibodies. The bactericidal activity of macrophages induced by IFN-γ were reported in mouse macrophages against *Toxoplasma* and in human monocyte-derived macrophages against *Chlamydia* (47).

The anti-viral activity of IFN-γ were evidenced in varicella zoster virus infection. There was enhanced survival of neurons that were infected with the virus after treatment as observed by Baird et al. (48) as IFN-γ inhibited the cytopathic effect and the virus’ DNA accumulation, transcription, and production. The proliferation of Hepatitis C virus in HIV+ patients were significantly halted by IFN-γ produced by NK cells in self-limited course of acute hepatitis C in HIV+ patients (49). The spontaneous clearance of HCV infection during the acute phase of hepatitis C was attributed to the robust IFN-γ-mediated inhibition of HCV replication by NK cells concluded Kokordelis et al. (49).

IFN-γ signaling also plays a role in T cell development where it facilitates Th1 development *via* induction of T-bet expression and suppression of GATA3 expression that drives Th2 differentiation. The development of Th17 cells is inhibited by IFN-γ as it inhibits the cytokines that promote the cells development (46). IFN-γ is involved in immunomodulation activity often related to type I interferon. IFN-γ or TNF-α can stimulate cells to synthesize proteosome subunits LMP2, LMP7 and MECL-1 to replace delta, MB1 and Z subunits where the catalytically active sites of the proteosome are found during proteosome neosynthesis. Proteosome is required for providing peptide ligands for the presentation on MHC class I molecules. The replacement from delta, MB1 and Z subunits to LMP2, LMP7 and MECL-1 subunits is believed to favor the production of peptide ligands of MHC class I molecules for the stimulation of cytotoxic T cells. This replacement also generated LMP2/LMP7/MECL-1- dependent epitopes in inflammatory sites. Benefits of this replacement include increased amount of class I MHC, increased binding strength between class I MHC and peptide fragments from pathogens, and increased epitopes diversity which allowed class I MHC to bind to a wider range of proteasome-derived peptides. Thus, higher levels of active class I MHC recognized by CD8+ T cell increases immune surveillance on inflammatory site (50). IFN-γ, added during the differentiation of dendritic cells, switches the differentiation pathway towards macrophages (47). Macrophage is one of the key cells needed in presenting antigens to helper T cells (51). The expression of MHC class II molecules can be augmented by IFN-γ as shown by Akbar et al. (52) in mice with subnormal expression of MHC class II molecules and in cultured human blood-derived dendritic cells by Rongcun et al. (53). The involvement of IFN-γ in enhancing the expression of MHC class II molecules was also reported by Thelemann et al. (54) in their adoptive transfer colitis model where IFN-γ was noted as the main cytokine that drove the MHC class II’s expression on intestinal epithelial cells and CD4^+^ T cells were the main source of the cytokine. This induction revealed the anti-inflammatory action of IFN-γ as there was reduction of colitogenic CD4^+^ T cells during chronic bacterial-driven colitis offering protection against colitis (54). IFN-γ is also needed in the optimal production of IL-12), an important marker for dendritic cell activation (47). IFN-γ protected bone marrow macrophage (BMM) from apoptosis as induced by the expression of p21^Waf1^ and stopped cell cycle at the G_1_/S boundary (55). Long survival time of macrophage allows prolonged stimulation of Th1 against inflammation as well as phagocytize pathogenic substance and repair tissue injury. Xaus et al. (55) observations also explained the role of IFN-γ in delayed hypersensitivity reaction.

#### 2.2.1 Pharmacotherapy applications of IFN-γ

IFN-γ is one of the most important cytokines in innate and adaptive immunity. Chronic granulomatous disease is an inherited disorder of phagocyte dysfunction. IFNγ-1b with similar biological activity as natural human IFN-γ can be given in various route of administration in treating this disorder. Intravenous administration has rapid drug clearance but reach peak plasma concentration in shortest time. Intramuscular injection has longer half-life followed by subcutaneous injection with increase of plasma concentration in more steady manner. Drug elimination occurs mostly in the biliary route. IFNγ-1b parenteral treatment mimics endogenous IFN-γ action in enhancement of phagocytosis and restoration of phagocyte NADPH oxidase system in patient with chronic granulomatous disease (56). Other than chronic granulomatous disease, IFN-γ immunotherapy is often used to treat cancer, tuberculosis, cystic fibrosis, hepatitis, osteoporosis, scleroderma, and invasive fungal infection. IFN-γ and interferon-inducible protein kinase (PKR) work hand in hand in the autophagy of osteosarcoma cells as shown by Xu et al. (57). Xu et al. (57) reported the molecular mechanisms of the anti-tumor effects of IFN-γ in osteosarcoma cells. They found that the cytokine causes the accumulation of autophagosomes in osteosarcoma cells and the conversion of autophagy marker light chain 3 (LC3)-I to LC3-II accompanied by puncta formation together with PKR. Owing to its immunomodulatory effects, IFN-γ have been used in treating immunodeficiency syndromes such as hyperimmunoglobulinemia E (hyper-IgE). Hyper-IgE patients suffer severe and recurrent *Staphylococcus aureus* infections and treatment with recombinant IFN-γ saw improvement in clinical symptoms of disease (58).

IFN-γ therapy has successfully reversed the damaging effects of fibrosis by restoring the total lung capacity and partial pressure of lung oxygen which also see the reduction of pro-inflammatory cytokines (58). IFN-γ in combination with conventional anti-microbials showed better outcome in treating visceral or cutaneous leishmaniasis (58). Badaro et al. (59) reported significant improvement in symptoms, measures of anemia and leukopenia, weight gain, decrease in spleen size and absence or reduced leishmania in splenic aspirates in 14 patients who underwent combination therapy of IFN-γ and pentavalent antimony. Aerosolized IFN-γ was shown to be a potential and effective prophylactic agent to treat multi-drug resistant tuberculosis (TB) patients who do not respond to other treatments. The alveolar macrophages of these patients showed higher activity and better effector responses after three times weekly treatment for 2 months. Even though reversion of sputum smears to positive was noted after therapy termination, it could still be used to treat TB patients in this category (58). **Table 3** summarized the numerous clinical trials of IFN-γ and their results.

**Table 3:** Clinical trials of IFN-γ and their results.

Identifier Number (Study Date)	Clinical Trial Title	Treatment Arms			Study Results	References
	The International Chronic Granulomatous Disease Cooperative Study Group: Randomized double masking clinical trials on IFNγ-1b in patients with chronic granulomatous disease	Participants received 50 μg/m^2^ of body surface area of interferon gamma (subcutaneous, thrice a week) for up to a year	Participants received 50 μg/m^2^ of body surface area of placebo (subcutaneous, thrice a week) for up to a year		77% of participants free from serious infection with IFNγ-1b SC three times a week for one year compared to 30% of participants free from serious infection with placebo.	(60)
NCT00201123 (April 2005 – August 2007)	Host Response to Tuberculosis and Acquired Immune Deficiency Syndrome	Participants received placebo standard treatment of isoniazid, rifampin, pyrazinamide anti-tuberculous therapy	Participants received aerosol interferon-gamma plus isoniazid, rifampin and pyrazinamide	Participants received subcutaneous interferon-gamma plus isoniazid, rifampin and pyrazinamide	60% of TB patients have sputum conversion with aerosol IFNγ-1b compared to 36% TB patients have sputum conversion with standard anti-tuberculosis medication.	(61)
NCT02584608 (January 1, 2016 – November 12, 2019)	Phase 2a Study of Interferon Gamma-1b for the Treatment of Autosomal Dominant Type 2 Osteopetrosis	Participants received 50 μg/m^2^ ACTIMMUNE (subcutaneous, three times per week) for 8 weeks			2.2% changes of beta-C-terminal telopeptide (CTX) and 2.1% type I collagen N-telopeptide (NTX) to creatinine ratio with ACTIMMUNE (IFNγ-1b) 50 µg/m2 SC three times per week for 8 weeks improves bone resorption markers from baseline to 14 weeks.	(62)

### 2.3 Type III interferons

IFN-λ 1-4 belong to the Type III family (9). Even though Type I and Type III IFN activate similar signaling pathways and induce similar ISGs, they have different viral infection-induced expression kinetics *in vivo*. Type I IFN are produced earlier than Type III IFNs, with its receptors expressed ubiquitously and trigger faster and stronger ISG induction. Type I IFN also promote additional expression of proinflammatory cytokines and chemokines leading to immunopathology where excessive responses of Type I IFN are unrestrained (9). Type III IFN bind to the heterodimeric receptor, IFNLR which is made up of IFNLR1 and IL10Rβ. They bind to IFNLR1 with high affinity and recruits IL10Rβ to form a signaling-competent ternary complex (63). Type III IFN on the other hand are produced later than Type I IFN, and with limited receptor expression that are confined to the epithelial cells and a subset of myeloid lineage leukocytes (9). Type III IFN also produce a less inflammatory and localized response than Type I IFN (64). Type III IFN responses are more restricted compared to Type I IFN. For example, mucosal epithelial tissues and intestinal epithelial cells of mice, and the lung epithelium to a certain extent respond to Type III IFN. Similarly, in humans, the mucosal epithelial tissues and liver respond to this IFN (65). Type III IFN are expressed in many primary human cell types of hematopoietic lineage and the nonhematopoietic cells that expressed these IFN are epithelial cells (65).

Type III IFN indirectly modulate T cell responses *via* DCs as there are limited number of IFNLR1 on T cells. Several groups found that by adding IFN-λ during peripheral blood mononuclear cell stimulation or mixed lymphocyte reaction reduced the production of Th2 cytokines while increasing the production of IFN-γ (63). Morrow et al. (66) showed that IFN-λ3 reduces regulatory T-cell populations, increase percentage of splenic CD8^+^ T cells in vaccinated animals during DNA vaccination. They also reported that IFN-λ3 as an adjuvant to DNA vaccine conferred 100% protection from mortality after a lethal influenza challenge (66). Researchers have reported mix effects of IFN-λs on B cell and antibody responses. In some instance, IFN-λ1 increases TLR-mediated activation of human B cells and IgG production while recombinant IFN-λ was found to inhibit IAV-stimulated Th2 cytokine release, B cell proliferation and production of antiviral IgG (63). Type III IFN can act directly on neutrophils which express IFN-λR1. Both interleukin-1 (IL-1) and interleukin-17 (IL-17) and neutrophil recruitment were suppressed in arthritis and other models of inflammation (65).

As an antiviral, Type III IFN successfully eliminated norovirus even without the adaptive immune system especially in the gastrointestinal tract (65). Though further studies are needed to support these early findings, Type III IFN may play a role in the vaginal epithelia during vaginal infection as reported by Ank et al. (67). Toll-like receptor 3 (TLR3) and toll-like receptor 9 (TLR9)-mediated antiviral defense were not expressed during vaginal infection with herpes simplex virus 2 in IFN-λR1 knockout mouse (67). Type III IFN has shown indirect effect against West Nile Virus where it limited neuroinvasion of the virus by acting upon the tight junction integrity of the mouse brain microvascular endothelial cells while maintaining the BBB (65).

Lasfar et al. (68) have previously compiled studies that showed the direct effects of type III IFN on cancer cells. It was proposed that type III IFN could offer a more targeted therapy as IFNLR1 are expressed in fewer cells and fewer cells respond to IFN-λ. Type III IFN acts by inhibiting the cancer cells from proliferating and promotes apoptosis of the cancerous cells (65). Several studies have shown that type III IFN inhibited the growth of several different cell lines and reduced local and metastatic tumor formation in mice from lung, liver, breast, and prostate cancers (65). Lasfar et al. (69) also found that type III IFN have indirect effect on tumors. They found suppressed tumor angiogenesis in a melanoma mouse model because of changes in the tumor microenvironment by IFN-λ. Additionally, they also found that T cell and NK cell responses to melanoma, lung adenocarcinoma and breast cancer were increased by IFN-λ (68).

Elevation of type III IFN also have been noted in several autoimmune diseases. Elevation of IFN-λ1 and ISG expression and upregulation of chemokines CXCL10 and CXCL11 were recorded in the skin lesions of psoriatic patients when compared to non-lesional skin from these patients or skin from healthy donors which do not have IFN-λ (63). IFN-λ mRNA and protein were also higher in systemic lupus erythematosus (SLE) patients with active arthritis and RA patients. On the other hand, IFN-λ therapy could also have anti-inflammatory effect as seen in collagen-induced arthritis. Pro-inflammatory Th17 and γδ T cells in the joints and inguinal lymph nodes of these mice were reduced with IFN-λ treatment (63). The clinical trials of IFN-λ and their results were summarized in **Table 4**.

**Table 4:** Clinical trials of IFN-λ and their results.

Identifier Number (Study Date)	Clinical Trial Title	Treatment Arms			Study Results	References
NCT02765802 (October 19, 2016 – December 12, 2018)	A Phase 2 Study to Evaluate the Safety, Tolerability and Pharmacodynamics of Pegylated Interferon Lambda Monotherapy in Patients with Chronic Hepatitis Delta Virus Infection (LIMT)	Participants received 180 μg peginterferon lambda-1A (subcutaneous, once weekly) for 48 weeks	Participants received 120 μg peginterferon lambda-1A (subcutaneous, once weekly) for 48 weeks		No results posted on ClinicalTrials.gov	(70)
NCT00565539 (December 2007 – October 2009)	A Phase 1 Study to Assess the Safety and Antiviral Activity of PEG-rIL-29 Administered as a Single Agent and in Combination with Ribavirin in Treatment-Relapsed and Treatment-Naïve Subjects with Chronic Hepatitis C Virus Infection	Participants received PEGylated recombinant interleukin 29 (PEG-rIL-29) (subcutaneous) weekly or every other week			No results posted on ClinicalTrials.gov	(71)
NCT01204762 (November 2010 – December 2013)	Dose-Ranging Study to Evaluate the Safety, Efficacy and Pharmacokinetics of Pegylated Interferon Lambda (BMS-914143) Monotherapy in Interferon-Naïve Patients with Chronic Hepatitis B Virus Infection who are HBeAg-positive	Participants received 180 μg pegIFN (subcutaneous, once weekly) for 48 weeks	Participants received 180 μg pegIFN-2a (subcutaneous, once weekly) for 48 weeks	Participants received 180 μg pegIFN lambda (subcutaneous, once weekly) for 48 weeks and 0.5 mg Entecavir tablet (oral, once daily, 12 weeks initial monotherapy followed by 48 weeks combination therapy with pegIFN lambda	No results posted on ClinicalTrials.gov	(72)

#### 2.3.1 Clinical applications of IFN-λ

As of 2019, there are no approved IFN-λ drug for human use even though pre-clinical studies have shown positive therapeutic benefits with reduced side effects experienced with Type I IFN according to Lazear et al. (63).

## 3 Role of neurotransmitters in IFN-induced mood dysregulation

Dopamine and serotonin (5-HT) are hormones that not only play a major role in mood and emotion regulations but also an individual’s physical health. They have been associated with certain biological pathways that led to mood dysregulation such as depression and suicidal thoughts (73). Kynurenine pathway has been alleged to be a contributing factor to mood dysregulation such as depression as it could reduce tryptophan’s availability since it is the main precursor for the synthesis of 5-HT. It has also been reported to possess an indirect action on *N*-methyl-D-aspartate (NMDA) receptor that leads to neurotoxicity effect (74).

Indoleamine 2,3-dioxgenase 1 (IDO1) is a gene that encodes the enzyme indoleamine 2,3-dioxygenase (IDO) that plays a part in the body immune system. Particularly, IDO1 is responsible for the metabolism of tryptophan (TRYP) to kynurenine (KYN). This typical step is the rate limiting step in the pathway which involves other two important enzymes – IDO2 and tryptophan 2,3-dioxygenase 2 (TDO2) that convert tryptophan to *N-*formylkynurenine before further metabolizing it into KYN (75). IFN-α is a ubiquitous signaling protein that stimulates the IDO activity, promoting the conversion of TRYP to KYN. At the same time, since TRYP is the precursor for 5-HT, there is negative action on the synthesis of 5-HT. Once the TRYP is shunted away from 5-HT, it will undergo further metabolism by kynurenine aminotransferase (KAT) into kynurenic acid. There is an alternative pathway of KYN which it will be converted into 3-hydroxykynurenine (3-OH-KYN) with the help of a catalyzing enzyme - kynurenine hydroxylase. Eventually, 3-OH-KYN will undergo several pathway metabolisms and be broken down into two end-product metabolites, namely quinolinic acid and picolinic acid. Quinolinic acid will then transform into a cofactor NAD. Wichers et al. (76) reported an elevation of kynurenine/kynurenic acid ratio during IFN-α therapy of HCV infected patient. Kynurenic acid is known to have antagonistic action on NMDA receptor since the synthesis of kynurenic acid prevents further metabolism into quinolinic acid. Moreover, KYN and kynurenic acid possess anti-inflammation effect while in contrast, quinolinic acid cause inflammation through its proinflammatory action. This effect can be seen through quinolinic acid which increases IFN-γ and decreases cytokine IL-10 (anti-inflammatory) levels. Interestingly, in astrocytes which have low concentration of kynurenine hydroxylase, higher ratio of tryptophan will be converted to kynurenic acid rather than quinolinic acid. Hence, inflammation induced by TRYP metabolites may be downregulated due to anti-inflammatory effect of kynurenic acid. Conversely, with macrophage, or microglia presence, which will convert KYN to quinolinic acid, the pro-inflammatory action will be initiated and further intensifies inflammation. Apart from being a NMDA receptor agonist, quinolinic acid can augment lipid peroxidation which will lead to cellular apoptosis while kynurenic acid has exactly the opposite effect to prevent lipid peroxidation (77). In short, when KYN is being converted into 3-OH-KYN, it will ultimately be involved in the activation of NMDA receptor that leads to neurotoxicity that is associated with diseases such as Alzheimer’s, amyotrophic lateral sclerosis (ALS), and depression (74). In fact, IFN-α stimulation that leads to degradation of serotonin is indirect through various cytokines such as TNF-α and IFN-γ. Baranyi et al. (74) proved that IDO activity increase can be seen through the intensification of KYN/TRYP ratio in patient receiving IFN-α therapy compared to baseline. Notably, Galvão-de Almeida et al. (78) conducted a cross-sectional study in Brazil to determine adverse effect related to different variation of IDO gene and their results claim that there is no association between IDO gene and IFN-α induced depression.

**Figure 1:**
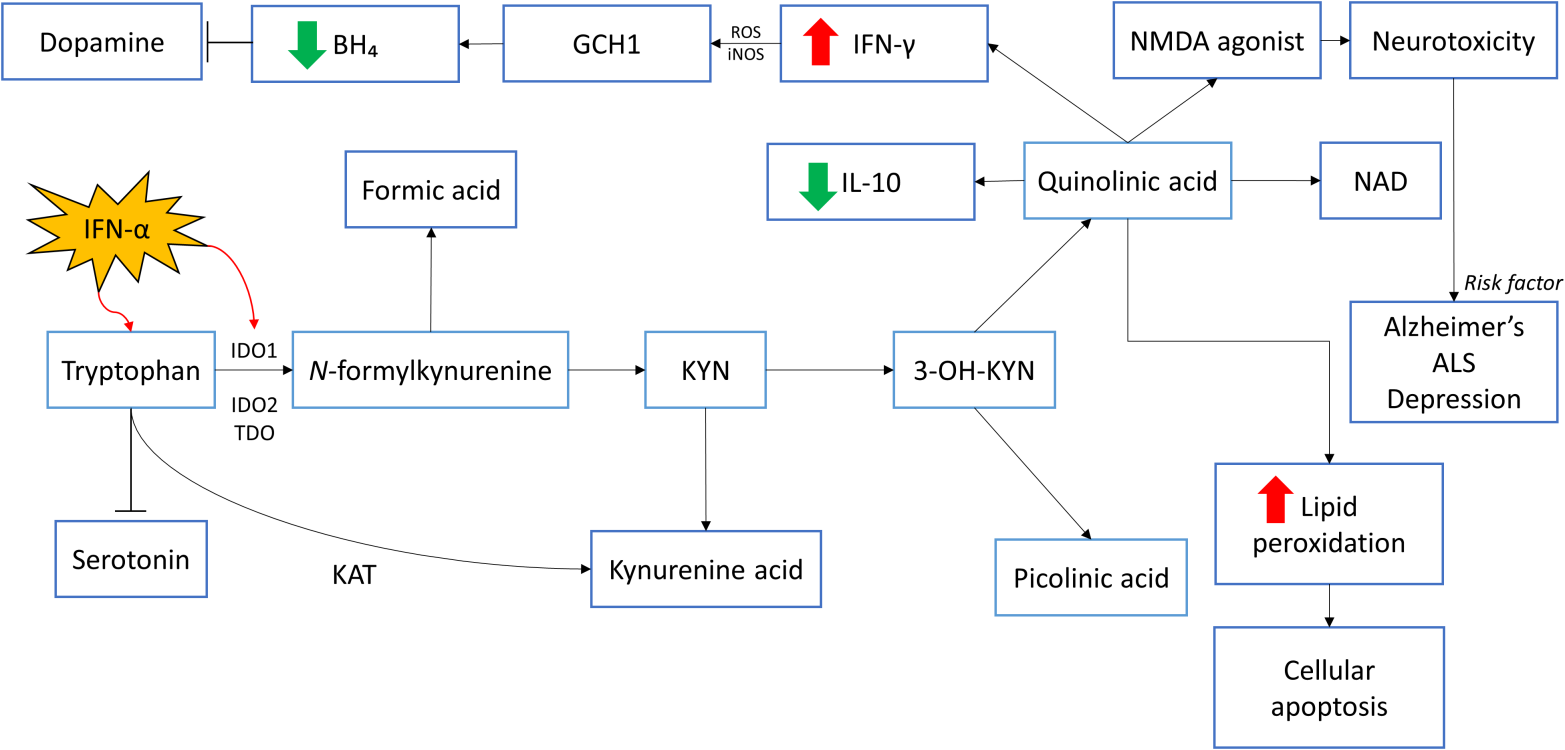
The roles of neurotransmitters in IFN-induced mood dysregulation. IFN-α stimulates IDO1 activity, stimulating the conversion of tryptophan to kynurenine and affects the synthesis of serotonin. Through several pathway metabolisms, kynurenine will be converted to quinolinic acid and picolinic acid. Quinolinic acid decreases the level of IL-10 which has anti-inflammatory effects. The negative effects of quinolinic acid include increasing the levels of IFN-γ which causes inflammation. This inflammatory response result in the production of ROS and iNOS which decreases BH₄. This eventually reduces dopamine secretion. Quinolinic acid is an NMDA agonist and can cause neurotoxicity as seen in patients with Alzheimer’s disease, ALS and depression. Quinolinic acid could cause cellular apoptosis too through the increase in lipid peroxidation. *IDO1: Indoleamine 2,3-dioxygenase 1; IDO2: Indoleamine 2,3-dioxygenase 2; TDO2: tryptophan 2,3-dioxygenase; KYN: Kynurenine; NAD: Nicotinamide adenine dinucleotide; NMDA: N-methyl-D-aspartate; IL-10: Interleukin-10; IFN-γ: Interferon-gamma; IFN-α: Interferon-alpha; ROS: Reactive oxygen species; iNOS: Inducible nitric oxide synthase; GCH1: GTP cyclohydrolase I; BH₄: Tetrahydrobiopterin; KAT: Kynurenine aminotransferase.

Besides KYN pathway that causes degradation in several neurotransmitter precursor, IFN-γ is found to be involved in the action of tetrahydrobiopterin (BH_4_). BH_4_ is a cofactor that is necessary for the biosynthesis of neurotransmitter such as dopamine, melatonin, 5-HT, etc., by converting respective amino acids to their precursors. Guanosine triphosphate cyclohydrolase-1 (GCH1) is an enzyme that catalyzes the formation of BH_4_ through conversion of GTP to dihydroneopterin triphosphate. The biosynthesis process of BH_4_ predominantly take place in dopaminergic and serotoninergic synaptosomes (79). The direct effect of BH_4_ on dopamine synthesis can be seen through its role as enzyme cofactor of phenylalanine hydroxylase (PAH) and tyrosine hydroxylase (TH) which is essential for the conversion of phenylalanine to tyrosine and tyrosine to levodopa (L-dopa) respectively before L-dopa converts into neurotransmitter dopamine. Of note, BH_4_ is also an important cofactor for the conversion of arginine to nitric oxide (NO) with the involvement of NO synthases (NOS). BH_4_ is then oxidized into 7,8-dihydroneopterin (BH_2_) and dihydroxanthoptein (XPH_2_) (13). Shoedon et al. have shown that when there is stimulation to the immune response, IFN-γ will induce GCH1 activity which will lead to increase in the synthesis of BH_4_ (79). However, these increased cytokines induced activity of GCH1 does not increase the production of 5-HT or dopamine since inflammation also induces the production of reactive oxygen species (ROS) and inducible NOS (iNOS). The consequences of elevated ROS and iNOS result in decrease of BH_4_ through the production of oxygen free radicals instead of NO. Evidence to this fact is the reduction in central nervous system (CNS) BH_4_ concentration after intramuscular injection of rat with IFN-α. Besides, further inhibition of iNOS increases the production of NO shown to overturn the outcomes of IFN-α (80). **Figure 1** provides a summary of the roles of neurotransmitters in IFN-induced mood dysregulation.

Some studies showed that quinolinic acid induces its toxicity through glutamatergic receptor in the brain. Glutamate is the most abundant excitatory neurotransmitter released upon stimulation by nerve impulse and exerts inotropic effect when it binds to specific receptor. To prevent the process of excitotoxicity with the accumulation of glutamate, sodium-dependent and sodium-independent glutamate uptake carriers are responsible to maintain glutamate at an optimum concentration (81, 82).

## 4 IFN-α and depression

The use of IFN-α as a therapeutic is seen in many diseases that include chronic HCV infection, HBV infection, melanoma, lymphoma, and AIDS-related Kaposi’s sarcoma (83). However, its adverse side effects outweigh its beneficial outcomes. It was reported that up to 50% of HCV-infected patients who underwent IFN-α therapy developed depression with 30% of them developing depression as early as the first 3 months of treatment initiation (83). Apart from that, patients also reported incidences of acute sickness, fatigue, malaise, myalgia, arthralgia, anorexia, apathy, and cognitive impairment stated (83, 84). IFN-α-related depression could be explained in several pathomechanisms which include its link to inflammation, neurotransmitter imbalance, neurodegeneration and neuroplasticity and oxidative stress (84).

**Figure 2:**
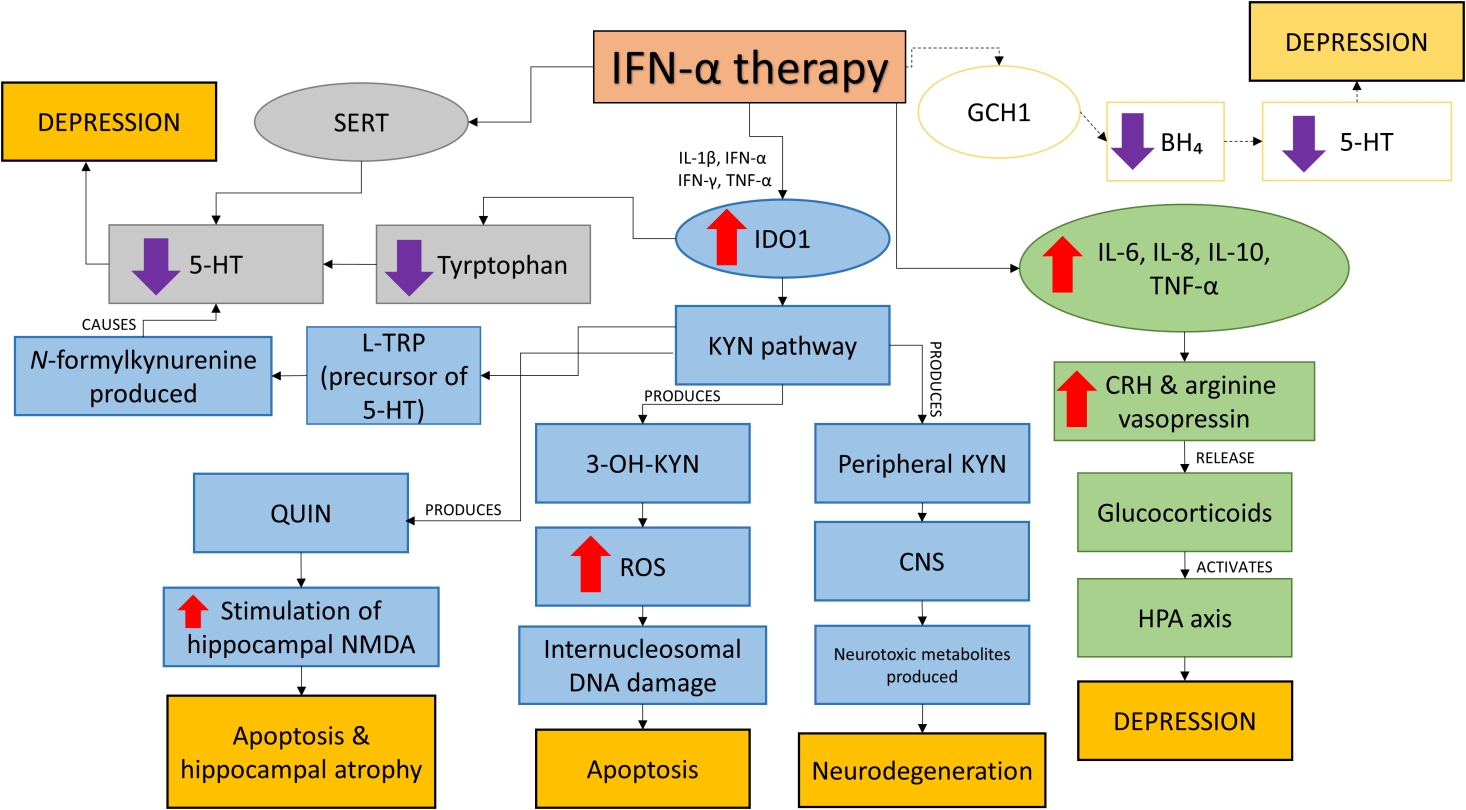
The effects of IFN-α therapy. IFN-α disrupts IDO1, SERT and possibly GCH1 systems and HPA axis leading to neurological changes and depression. Lower 5-HT production which is linked to depression is caused by the disruption on the SERT and IDO1 systems. IFN- also causes apoptosis, hippocampal atrophy and neurodegeneration *via* the KYN pathway as a result of increased IDO1 by (i) inhibiting the conversion of L-TRP, precursor of 5-HT (ii) production of QUIN which increases the stimulation of hippocampal NMDA (iii) production of 3-OH-KYN leading to increased ROS production causing intranucleosomal DNA damages and (iv) production of peripheral KYN which affects the CNS and subsequently neurodegeneration due to the release of neurotoxic metabolites. IFN- therapy also increases the production of pro-inflammatory cytokines which activate the HPA axis and causes depression. Another possible target of IFN-α is on GCH1 which reduces BH4 production, reducing 5-HT and leads to depression. *IFN-α, Interferon-alpha; IL-1β, Interleukin-1beta; IFN-γ, Interferon-gamma; TNF-α, TNF-alpha; 5-HT, Serotonin; SERT, Serotonin transporter; IDO1, Indoleamine 2,3 dioxygenase; KYN, Kynurenine; 3-OH-KYN, 3-hydroxy-kynurenine; QUIN, Quinolinic acid; NMDA, N-methyl-D-aspartate; CNS, Central nervous system; ROS, Reactive oxygen species; BH₄, Tetrahydrobiopterin; CRH, Corticotropin-releasing hormone; HPA, Hypothalamic-pituitary-adrenal; GCH1, GTP cyclohydrolase I*.

Deregulation of neurotransmitter systems – 5-HT, dopamine and glutamate were noted in major depressive disorder. IFN-α causes disruptions to these systems by disrupting the expression; activation of ubiquitous indoleamine 2,3 dioxygenase (IDO1) and inducing the 5-HT transporter serotonin transporter (SERT); and inducing (GCH1). The summary of the effects of IFN-α therapy is shown in **Figure 2**.

The biosynthesis of 5-HT is reduced as a result of increased in IDO1 activity which reduces the availability of tryptophan (84). This phenomenon is induced by pro-inflammatory cytokines IL-1β, IFN-α, IFN-γ and TNF-α (83). The activation of IDO1 also affects the kynurenine (KYN) pathway as IDO1 is the rate-limiting enzyme of the pathway and it converts L-TRP, precursor of 5-HT to form *N-*formylkynurenine (85). A drop in TRP levels were seen in HCV patients taking IFN-α therapy within 4 to 6 months after the initiation of therapy and higher kynurenine plasma levels were recorded at week 2 of treatment (which remained the same at week 4, 16 and 24) reported Bonaccorso et al. (86). The kynurenine pathway will be activated by IFN-α due to upregulated expression of IDO. A variety of neuroactive metabolites such as 3-hydroxy-kynurenine (3-OH-KYN) and quinolinic acid (QUIN) will be produced (85). Peripheral kynurenine can easily affect the central nervous system as it is transported through the BBB by a large neutral amino acid carrier and in the brain, it will be taken up by glia cells to be metabolized. This is how neurotoxic metabolites are formed in the brain causing neurodegeneration (85). QUIN may increase the stimulation of hippocampal NMDA receptors leading to apoptosis and hippocampal atrophy (85). Elevation of QUIN is reported in the cingulate cortex of suicidal patients with acute idiopathic depression (84). QUIN also was shown to induce anxiety- and depression-like behavior in rats and mice by Vecsei and Beal (87) and Lapin et al. (88). Maes et al. (77) reported that QUIN has pro-inflammatory effects as they saw an increase of plasma ratio of pro-inflammatory IFN-γ to anti-inflammatory IL-10 in their model of LPS-induced immune activation in healthy humans. Subsequently, they also discovered that elevated levels of QUIN increase oxidative stress as more free oxygen radical species were formed (89). This generated free radical species causes QUIN-induced lipid peroxidation in membrane lipids and proteins changing neuronal membrane fluidity, receptor function and ion permeability (85). Another kynurenine metabolite produced is 3-OH-KYN which increases the production of reactive oxygen species (ROS) after interacting with cellular xanthine oxidase (85). The ROS produced induce internucleosomal DNA cleavage causing apoptosis. Relatively low levels of 3-OH-KYN cause neurotoxicity and together with QUIN, free radicals will be produced in large amount (85). Thus, IFN-α-induced depression is a result of the cytokine’s action on 5-HT brain neurotransmission caused by pro-inflammatory cytokines and the induction of IDO.

The availability of 5-HT in the extracellular space is also affected by the induction of SERT by IFN-α. By inducing SERT, IFN-α reduces the levels of 5-HT in the synaptic clefts as it promotes the reuptake of 5-HT from the extracellular space (84). This event is dependent on the mitogen-activated protein kinase (MAPK) p38, extracellular regulated kinase (ERK)-1/2 and Jun kinase (JNK)-1/2 signaling pathways as found by Tsao et al. (90). Though there is a lack of supportive data of the involvement of GCH1 in IFN-α-related depression, its involvement is still considered important as demonstrated in the CSF of patients with chronic HCV infection. The CSF of these patients undergoing IFN-α therapy were presented with increased (BH_2_) and reduced (BH_4_) concentrations (84). BH_4_ is needed in 5-HT and dopamine biosynthesis as it is a cofactor of the rate limiting enzyme phenylalanine hydroxylase thus affecting 5-HT and dopamine biosynthesis explained Hoyo-Becerra et al. (84).

Cytokines such as IL-6 and TNF-α are known to be involved in IFN-α-induced depression and high levels of these cytokines is associated with increased risk of major depressive disorder (91). Using a consolidated approach to investigate the pathogenesis of psychiatric disorder with peripheral blood to measure gene expression (mRNA) levels, Hepgul et al. (91) found 73 genes (at baseline i.e. before treatment) that were differentially expressed in HCV patients who developed depression after IFN-α treatment. Subsequently after 4 weeks of treatment they found modulation of 592 genes in this group of patients and these genes are linked to inflammation, neuroplasticity and oxidative stress pathways. The same results were obtained at week 24 of the treatment. Through their observations, Hepgul et al. (91) concluded that patients who developed depression because of IFN-α treatment are more sensitive towards IFN-α. IL-6, TNF-α and IL-1, and their association to depression could be linked to their ability to trigger the activation of HPA axis. This was done through the activation of corticotropin-releasing hormone (CRH) and arginine vasopressin which releases glucocorticoids (92). Coincidentally, hyperactivity of the HPA axis is linked to depression; depressed patients showed elevation of CRH concentrations in their cerebrospinal fluid (CSF) (92). In their study, Wichers et al. (92) found positive correlation between the high levels of soluble interleukin-2 receptor (sIL2-r), TNF-α and IL-6 and depressive symptoms in patients with active chronic HCV infection receiving IFN-α treatment. Their findings echoed Bonaccorso et al.’s findings whereby IL-6, IL-8 and IL-10 were in high amounts during IFN-α treatment (92). However, conflicting results were seen in Capuron et al.’s study in 2003 as they found no correlation between IL-6 and depression in patients with malignant melanoma undergoing IFN-α treatment. Similarly, they reported significant increase in adrenocorticotropic hormone and cortisol responses while Wichers et al. (92) did not. This could possibly be due to the different disease profile of the subjects as Raison et al. (93) found increase of IL-6 and monocyte chemoattractant protein-1 in the CSF of IFN-α treated patients but no elevation in plasma IL-6. The evidences of IFN-α-induced depression in IFN-α treated patients infected with HCV and cancer were presented in **Table 5** while **Figure 2** summarizes the effects of IFN-α on depression.

**Table 5:** IFN-α treatment and depression in patients with HCV and cancer.

Disease	Observation Period	Number of Patients	Observations		Reference
			Symptoms	Onset of Symptom(s)	
Chronic viral hepatitis	Four-to-12-month course of recombinant human IFN-α therapy	58	17% of patients experienced one or more of the following symptoms: irritability, mood swing, depression, tearfulness, lack of awareness, agitation, paranoia, and suicidal ideation	Symptoms started 1 to 3 months after the initiation of therapy	Renault et al. (94)
Chronic active hepatitis C	Patients were assessed before and 2, 4, 12 and 24 weeks after therapy initiation	85	37% of patients without depression developed a major depressive episode at least once during therapy 5.9% stopped therapy due to physical side effects 4.7% stopped therapy due to depression	Depression diagnosed after 4 weeks of therapy have more severe depressive episode than before 4 weeks of therapy	Otsubo et al. (95)
Hepatitis C	Patients monitored weekly	39	33% of patients developed IFN-α-induced major depressive disorder.	Patients developed IFNα-induced depression after 12.1 weeks (average) of therapy	Hauser et al. (96)
Chronic active Hepatitis C	Patients evaluated at baseline and 3 months after therapy	30	40.7% of patients experienced following symptoms: sadness, irritability, insomnia, loss of appetite, and lack of energy.	Patients developed IFNα-induced depression after 3 months of therapy	Bonaccorso et al. (86)
Chronic Hepatitis C	Patients evaluated before therapy and once every 4 weeks during both 24-week treatment and 12 weeks after end of treatment	99	23.2% of patients developed IFN-α-induced major depressive disorder.	73.9% of patients developed IFN-α-induced depression within 8 weeks of therapy	Horikawa et al. (97)
Chronic Hepatitis C	Patients evaluated received IFN-α treatment for 12 months or no treatment	104	57.7% of patients experienced depression, anxiety or anger/hostility.		Kraus et al. (98)
Chronic Hepatitis C	Patients followed for 24 weeks; comparisons for 3 treatment groups were evaluated at baseline and at 4, 8, 12 and 24 weeks; untreated group only at baseline and at 12 and 24 weeks		26% patients receiving psychiatric treatment at baseline became more symptomatic during therapy 48% of patients not on neuropsychiatric treatment at baseline required treatment during therapy; 23% met criteria for major depression Control group showed little change over 24-week period	Changes in Beck Depression Inventory Scores were observed at week 4	Dieperink et al. (99)
Chronic Hepatitis C	Patients were observed for 24 weeks	55	27% had suicidal ideation while not on IFN therapy 43% had suicidal ideation in the IFN treated group		Dieperink et al. (100)
Hepatitis C	Patients evaluated at study enrolment and during treatment for 24 weeks	163 (74 with Hepatitis C; 37 with Hepatitis C and HIV; 76% were injecting drug users; 52 did not received treatment)	16% had depression at enrolment 35% developed new-onset depression during treatment		Alavi et al. (101)
Chronic myelogenous leukemia		25	Patients experienced following symptoms: depression, increased somatic concern, and stress reaction; below expectation on tests of cognitive speed, verbal memory and executive functions		Pavol et al. (102)
Melanoma	Patients evaluated at baseline and after 1, 3, 6 and 12 months after therapy	113	8 patients in IFN-α therapy group had significant degree of action tremor Greater increase in anxiety in treatment group		Caraceni et al. (103)
Melanoma		4	Patients developed depression or mania during therapy	Psychiatric side effects developed after 1 to 5 months of therapy	Greenberg et al. (104)

### 4.1 Regulation of hypothalamic-pituitary-adrenal (HPA) Axis in IFN-α Therapy

The HPA axis is an important yet complex pathway that plays a vital role in the maintenance of physiological homeostasis in the body in a stress situation (105). Optimum level of glucocorticoids is important in maintaining a structurally and functionally stable neuron. There are studies that suggest the profound role of glucocorticoids in the regulation of HPA axis through its involvement in negative feedback actions; any impairment to its function will lead to overstimulation of HPA axis (106). In response to stress, hypothalamus secrete CRH. The signal then induces the release of adrenocorticotropic hormone (ACTH) from the anterior pituitary gland which results in the production of circulatory cortisol hormone in the blood (107). Cortisol is the main glucocorticoid released from the adrenal cortex (108). It is a stress hormone that is released during stress or “fight-or-flight” situations. High concentration of cortisol initiates negative feedback loop which suppresses further release of CRH and ACTH (107). Cortisol is the key hormone in HPA axis which functions to lower down immune response by utilizing non-carbohydrate molecules for the synthesis of glucose through gluconeogenesis and decreasing inflammatory action (109). Although cortisol can be considered to have protective effect to reduce inflammation, long-term exposure to this hormone will lead to irreversible alteration to the neuronal system and function (110).

Apart from glucocorticoids, medial hypothalamic neurons are also involved in controlling the level of cortisol by mediating the action of CRH and vasopressin release. Of note, action of medial hypothalamic neurons is exerted through the action of endocannabinoids to cannabinoid receptor 1 (CB1) before leading to suppression of prefrontal cortex and hippocampus neurotransmitter release. Studies have found that long term exposure to stressors will result in impairment of CB1 receptor which in turn stimulate the HPA axis pathway (111). Joseph and Whirledge (112) claimed that upregulation of HPA-axis will have detrimental effects on CNS and it is associated with the pathogenesis of mood dysregulation.

Constant stimulation of HPA axis is known to result in an increase in neuronal activation in hippocampus, amygdala, and frontal cortex. This action plays a part in suppressing neuronal plasticity and neurogenesis which is highly associated with occurrence of depressive-like syndrome in cognitive impaired individuals (107). Cognitive impairment is one of the consequences of high level of cortisol levels due to dysregulation of HPA axis. This can be seen by the fact that decrease in depressive-like syndrome when the corticosterone synthesis has been suppressed during long term exposure of mild stress (113). In addition, hyperactivation of HPA axis will cause high level of adrenal gland secretion which impairs hippocampus leading to depressive behavior. During stress, the body responds to this phenomenon by secreting cortisol. Hypercortisolism is a stage cannot be prevented especially for individuals that have major depressive disorder (MDD) in which their body will have substantially high level of cortisol as reported by Bertollo et al. (114) in their cross-sectional study. One of the common methods to assess the effect of HPA-axis dysregulation on contribution to mood dysregulation is by administering exogenous corticosterone (CORT). Most of the studies have found that long term exposure of exogenous corticosterone leads to cognitive dysfunction and affected memory task of the hippocampus (115). From the neurobiological point of view, researchers have claimed that there is a decrease in hippocampal volume, alteration of prefrontal cortex and amygdala dendrites hypertrophy after administration of CORT (116).

**Figure 3:**
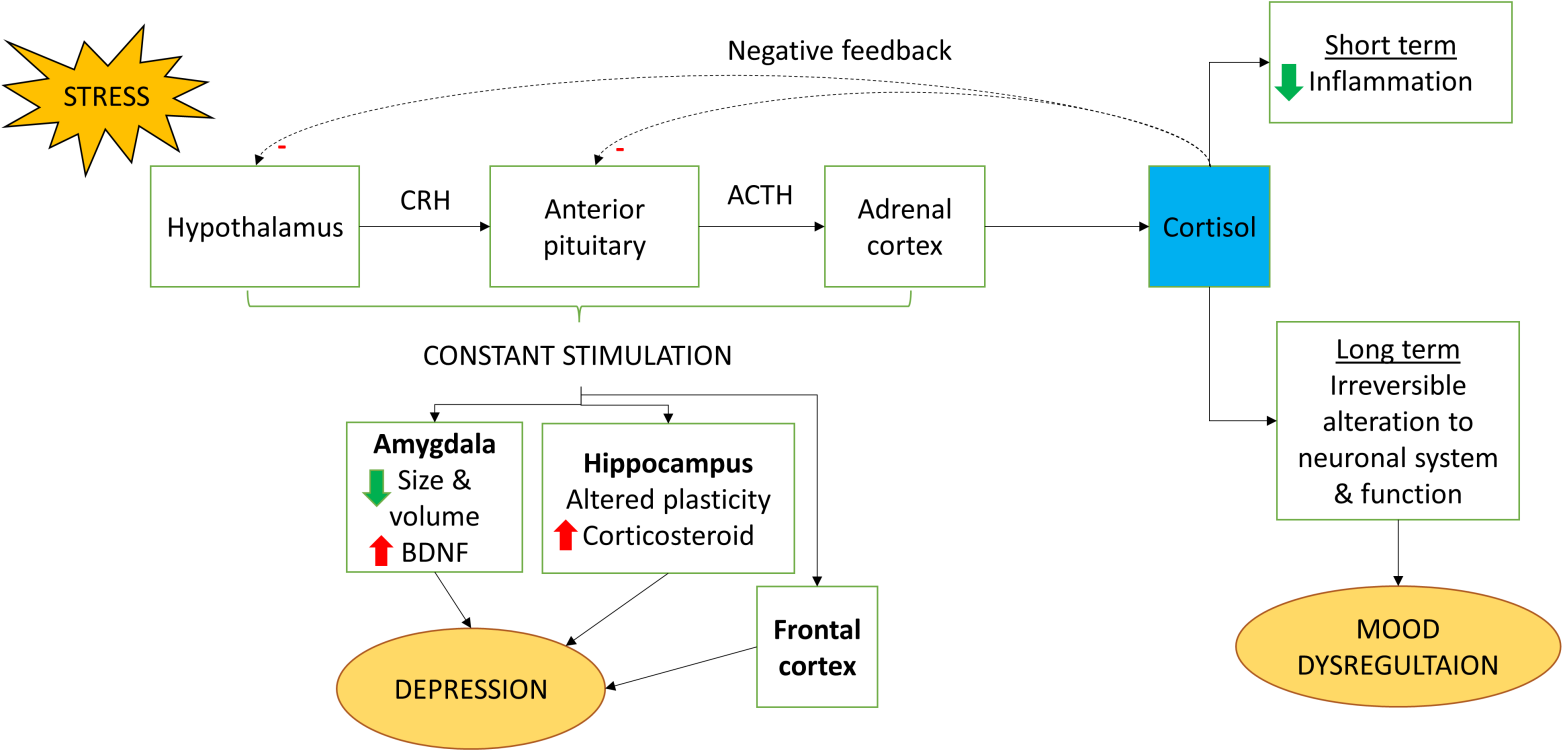
Regulation of HPA axis by IFN-α. When stimulated by stress, cortisol is being released by the HPA axis. Cortisol has short term benefit whereby it could reduce inflammation. However, long term exposure could lead to irreversible change to the neuronal system and function which causes mood dysregulation. Constant stimulation of the HPA axis also could lead to depression because of increased stimulation on the amygdala, hippocampus and frontal cortex. *CRH, Corticotropin-releasing hormone; ACTH, Adrenocorticotropic hormone*.

Amygdala and hippocampus are highly associated with the development of depressive-like behavior. Amygdala plays a role in cortical arousal and regulation of emotional stimuli. Evidence from a meta-analysis indicated that there are morphological changes to the amygdala especially the size and volume reduction in depressive individuals noting an increase in activity of brain-derived neurotrophic factor (BDNF) (117). BDNF is a protein which is important not only for the growth, maturation, and survival of neuron in the brain but also the regulation of synaptic transmission (118). Consequently, amygdala’s hyperactivity from negative stimuli can also be an early sign of depression (117, 119). Hippocampus’ profound role is well defined in learning and memory processing through neurogenesis. Since hippocampus and HPA axis are highly related to each other, stress will alter the degree of plasticity which will then lead to increase in the secretion of corticosteroid. Studies on rats have found that stress will have negative consequences on the hippocampal plasticity especially alteration in the volume and dysregulation of neurogenesis. The dysregulation of HPA axis in IFN-α therapy is shown in **Figure 3**.

## 5 Conclusion

IFN therapy has proven to be highly beneficial in addressing many diseases such as hepatitis, cancer, and multi-drug diseases. However, the therapy also brings about many neuropsychological disorders such as depression, anxiety, suicidal incidences, and other mild symptoms that affects the patients’ quality of life. Due to these disruptions, often patients decide to cease treatment earlier than the recommended treatment duration which impedes the treatment objective. Nevertheless, with proper management in terms of dose adjustment, patient monitoring and early intervention, the benefits of IFN therapy surpass its side effects. We hope that by highlighting this major side effect of IFN therapy, clinicians will be able to re-evaluate the pros and cons of this treatment option. On the other hand, adjunct therapy which may reduce the neuropsychological side effects of IFN therapy may be explored in the future.

## Author contributions

Conceptualization, MTL; Formal Analysis, JYL, JXH, ASFK, GL; Investigation, JYL, JXH, ASFK, GL; Writing – Original Draft Preparation, JYL, JXH and GL; Writing – Review and Editing, CLT, ASFK, Y-CH and MTL; Supervision, CLT and MTL; Funding Acquisition, Y-CH, CLT, ASFK and MTL. All authors contributed to the article and approved the submitted version.

## References

[1] Felger JC. Role of inflammation in depression and treatment implications. Handb Exp Pharmacology (2019) 250:255–86. doi: 10.1007/164_2018_166 30368652

[2] Rossi S Studer V Motta C Polidoro S Perugini J Macchiarulo G. ‘Neuroinflammation drives anxiety and depression in relapsing-remitting multiple sclerosis’. Neurology (2017) 89(13):1338–47. doi: 10.1212/WNL.0000000000004411 28842450

[3] Zheng Z-H Tu J-L Li X-H Hua Q Liu W-Z Liu Y. Neuroinflammation induces anxiety- and depressive-like behavior by modulating neuronal plasticity in the basolateral amygdala. Brain Behavior Immun (2021) 91:505–18. doi: 10.1016/j.bbi.2020.11.007 33161163

[4] Hepgul N Pariante CM Baraldi S Borsini A Bufalino C Russell A. Depression and anxiety in patients receiving interferon-alpha: The role of illness perceptions. J Health Psychol (2018) 23(11):1405–14. doi: 10.1177/1359105316658967 27458106

[5] Borsini A Cattaneo A Malpighi C Thuret S Harrison NA Consortium MRCI. ‘Interferon-alpha reduces human hippocampal neurogenesis and increases apoptosis *via* activation of distinct STAT1-dependent mechanisms’. Int J Neuropsychopharmacol (2018) 21(2):187–200. doi: 10.1093/ijnp/pyx083 29040650 PMC5793815

[6] Capuron L Neurauter G Musselman DL Lawson DH Nemeroff CB Fuchs D. ‘Interferon-alpha-induced changes in tryptophan metabolism. relationship to depression and paroxetine treatment’. Biol Psychiatry (2003) 54(9):906–14. doi: 10.1016/s0006-3223(03)00173-2 14573318

[7] Gisslinger H Svoboda T Clodi M Gilly B Ludwig H Havelec L. ‘Interferon-alpha stimulates the hypothalamic-pituitary-adrenal axis *in vivo* and *in vitro*’. Neuroendocrinology (1993) 57(3):489–95. doi: 10.1159/000126396 8391662

[8] Lucaciu LA Dumitrascu DL. ‘Depression and suicide ideation in chronic hepatitis c patients untreated and treated with interferon: prevalence, prevention, and treatment’. Ann Gastroenterol (2015) 28(4):440–7. PMC4585389 26424594

[9] Kim YM Shin EC. Type I and III interferon responses in SARS-CoV-2 infection. Exp Mol Med (2021) 53:750–60. doi: 10.1038/s12276-021-00592-0 PMC8099704 33953323

[10] Durbin RK Kotenko SV Durbin JE. ‘Interferon induction and function at the mucosal surface’. Immunol Rev (2013) 255(1):25–39. doi: 10.1111/imr.12101 23947345 PMC5972370

[11] van Boxel-Dezaire AH Rani MR Stark GR. ‘Complex modulation of cell type-specific signaling in response to type I interferons’. Immunity (2006) 25(3):361–72. doi: 10.1016/j.immuni.2006.08.014 16979568

[12] Perry A Chen G Zheng D Tang H Cheng G. The host type I interferon response to viral and bacterial infections. Cell Res (2005) 15:407–22. doi: 10.1038/sj.cr.7290309 15987599

[13] Felger JC Miller AH. ‘Cytokine effects on the basal ganglia and dopamine function: the subcortical source of inflammatory malaise’. Front Neuroendocrinol (2012) 33(3):315–27. doi: 10.1016/j.yfrne.2012.09.003 PMC3484236 23000204

[14] Mattei F Bracci L Tough DF Belardelli F Schiavoni G. ‘Type I IFN regulate DC turnover *in vivo*’. Eur J Immunol (2009) 39(7):1807–18. doi: 10.1002/eji.200939233 19544312

[15] Axtell RC Raman C Steinman L. ‘Type I interferons: beneficial in Th1 and detrimental in Th17 autoimmunity’. Clin Rev Allergy Immunol (2013) 44(2):114–20. doi: 10.1007/s12016-011-8296-5 PMC5478162 22231516

[16] Gibbert K Schlaak JF Yang D Dittmer U. ‘IFN-α subtypes: Distinct biological activities in anti-viral therapy’. Br J Pharmacol (2013) 168(5):1048–58. doi: 10.1111/bph.12010 PMC3594665 23072338

[17] Foster GR. Review article: Pegylated interferons: Chemical and clinical differences. Alimentary Pharmacol Ther (2004) 20(8):825–30. doi: 10.1111/j.1365-2036.2004.02170.x 15479353

[18] Rong L Perelson AS. ‘Treatment of hepatitis c virus infection with interferon and small molecule direct antivirals: viral kinetics and modeling’. Crit Rev Immunol (2010) 30(2):131–48. doi: 10.1615/critrevimmunol.v30.i2.30 PMC2882097 20370626

[19] Thyrell L Erickson S Zhivotovsky B Pokrovskaja K Sangfelt O Castro J. ‘Mechanisms of interferon-alpha induced apoptosis in malignant cells’. Oncogene (2002) 21(8):1251–62. doi: 10.1038/sj.onc.1205179 11850845

[20] Indraccolo S. Interferon-alpha as angiogenesis inhibitor: Learning from tumor models. Autoimmunity (2010) 43(3):244–7. doi: 10.3109/08916930903510963 20166871

[21] Tankurt E Tunca M Akbaylar H Gönen O. ‘Resolving familial Mediterranean fever attacks with interferon alpha’. Br J Rheumatol (1996) 35(11):1188–9. doi: 10.1093/rheumatology/35.11.1188 8948315

[22] Tunca M Tankurt E Akbaylar Akpinar H Akar S Hizli N Gönen O. ‘The efficacy of interferon alpha on colchicine-resistant familial Mediterranean fever attacks: a pilot study’. Br J Rheumatol (1997) 36(9):1005–8. doi: 10.1093/rheumatology/36.9.1005 9376975

[23] Tunca M Akar S Soytürk M Kirkali G Resmi H Akhunlar H. ‘The effect of interferon alpha administration on acute attacks of familial Mediterranean fever: A double-blind, placebo-controlled trial’. Clin Exp Rheumatol (2004) 22:S37–40. 15515782

[24] Tweezer-Zaks N Rabinovich E Lidar M Livneh A. Interferon-alpha as a treatment modality for colchicine- resistant familial Mediterranean fever. J Rheumatol (2008) 35(7):1362–5. 18528960

[25] Markowitz CE. ‘Interferon-beta’. Neurology (2007) 68(24 suppl 4):S8–S11. doi: 10.1212/01.wnl.0000277703.74115.d2 17562848

[26] Friedman RM. Clinical uses of interferons. Br J Clin Pharmacol (2008) 65:158–62. doi: 10.1111/j.1365-2125.2007.03055.x PMC2253698 18070219

[27] Haji Abdolvahab M Mofrad MR Schellekens H. ‘Interferon beta: From molecular level to therapeutic effects’. Int Rev Cell Mol Biol (2016) 326:343–72. doi: 10.1016/bs.ircmb.2016.06.001 27572132

[28] Yen J-H Kong W Ganea D. ‘IFN-β inhibits dendritic cell migration through STAT-1–mediated transcriptional suppression of CCR7 and matrix metalloproteinase 9’. J Immunol (2010) 184(7):3478 LP – 3486. doi: 10.4049/jimmunol.0902542 PMC2877494 20190134

[29] Kappos L Traboulsee A Constantinescu C Erälinna J-P Forrestal F Jongen P. ‘Long-term subcutaneous interferon beta-1a therapy in patients with relapsing-remitting MS’. Neurology (2006) 67(6):944 LP – 953. doi: 10.1212/01.wnl.0000237994.95410.ce 17000959

[30] Vervoordeldonk MJ Aalbers CJ Tak PP. ‘Interferon beta for rheumatoid arthritis: New clothes for an old kid on the block’. Ann Rheumatic Dis (2009) 68(2):157–8. doi: 10.1136/ard.2008.097899 19139202

[31] Yang X Zhang X Fu ML Weichselbaum RR Gajewski TF Guo Y. ‘Targeting the tumor microenvironment with interferon-β bridges innate and adaptive immune responses’. Cancer Cell (2014) 25(1):37–48. doi: 10.1016/j.ccr.2013.12.004 24434209 PMC3927846

[32] McCarthy SDS Majchrzak-Kita B Racine T Kozlowski HN Baker DP Hoenen T. ‘A rapid screening assay identifies monotherapy with interferon-ß and combination therapies with nucleoside analogs as effective inhibitors of Ebola virus’. PLoS Negl Trop Dis (2016) 10(1):e0004364. doi: 10.1371/journal.pntd.0004364 26752302 PMC4709101

[33] Smith LM Hensley LE Geisbert TW Johnson J Stossel A Honko A. ‘Interferon-β therapy prolongs survival in rhesus macaque models of Ebola and marburg hemorrhagic fever’. J Infect Dis (2013) 208(2):310–8. doi: 10.1093/infdis/jis921 PMC3685222 23255566

[34] Anon. Efficacy and safety of PEG-interferon alpha-2a plus ribavirin in genotype 1 chronic hepatitis c participants co-infected with human immunodeficiency virus. Available at: https://clinicaltrials.gov/ct2/show/study/NCT02761629.

[35] Anon. Pegylated interferon alfa-2a plus low dose ribavirin for treatment-naïve hemodialysis patients with chronic hepatitis c. Available at: https://www.clinicaltrials.gov/ct2/show/study/NCT00491244.

[36] Anon. A study of pegylated interferon alfa-2a and lamivudine in patients with hbeag-negative chronic hepatitis b virus (HBV). Available at: https://clinicaltrials.gov/ct2/show/NCT01095835.

[37] Anon. A study of pegylated interferon alfa-2a in combination with lamivudine or entecavir compared with untreated control group in children with hepatitis b envelope antigen (hbeag)-positive chronic hepatitis b (CHB) in the immune-tolerant phase. Available at: https://clinicaltrials.gov/ct2/show/study/NCT02263079.

[38] Anon. Antiviral activity of PEG-IFN-alpha-2A in chronic HIV-1 infection. Available at: https://clinicaltrials.gov/ct2/show/study/NCT00594880.

[39] Anon. Safety and efficacy of bevacizumab plus RAD001 versus interferon alfa-2a and bevacizumab for the first-line treatment in adult patients with kidney cancer. Available at: https://clinicaltrials.gov/ct2/show/NCT00719264.

[40] Anon. Temozolomide alone or with pegylated interferon-alpha 2b (PGI) in melanoma patients. Available at: https://www.clinicaltrials.gov/ct2/show/NCT00525031.

[41] Anon. Efficacy and safety study of peginterferon beta-1a in participants with relapsing multiple sclerosis - full text view. efficacy and safety study of peginterferon beta-1a in participants with relapsing multiple sclerosis. Available at: https://clinicaltrials.gov/ct2/show/study/NCT00906399.

[42] Anon. Long-term safety and efficacy study of peginterferon beta-1a - full text view. long-term safety and efficacy study of peginterferon beta-1a. Available at: https://clinicaltrials.gov/ct2/show/study/NCT01332019.

[43] Anon. A study comparing the effectiveness and safety of teriflunomide and interferon beta-1a in patients with relapsing multiple sclerosis - full text view. a study comparing the effectiveness and safety of teriflunomide and interferon beta-1a in patients with relapsing multiple sclerosis. Available at: https://clinicaltrials.gov/ct2/show/NCT00883337.

[44] Anon. A phase 2 study of interferon beta-1a (Avonex®) in ulcerative colitis. Available at: https://www.clinicaltrials.gov/ct2/show/NCT00616434.

[45] Anon. Interferon-beta1a (AVONEX) treatment of ulcerative colitis. Available at: https://clinicaltrials.gov/ct2/show/NCT00048347.

[46] Lin F Young H. The talented interferon-gamma. Adv Bioscience Biotechnol (2013) 4:6–13. doi: 10.4236/abb.2013.47A3002

[47] Billiau A Matthys P. ‘Interferon-gamma: A historical perspective’. Cytokine Growth Factor Rev (2009) 20(2):97–113. doi: 10.1016/j.cytogfr.2009.02.004 19268625

[48] Baird NL Bowlin JL Hotz TJ Cohrs RJ Gilden D Sandri-goldin RM. ‘Interferon gamma prolongs survival of varicella-zoster virus-infected human neurons *in vitro*’. J Virol (2015) 89(14):7425–7. doi: 10.1128/JVI.00594-15 PMC4473560 25948748

[49] Kokordelis P Krämer B Körner C Boesecke C Voigt E Ingiliz P. ‘An effective interferon-gamma-mediated inhibition of hepatitis c virus replication by natural killer cells is associated with spontaneous clearance of acute hepatitis c in human immunodeficiency virus-positive patients’. Hepatology (2014) 59(3):814–27. doi: 10.1002/hep.26782 24382664

[50] Groettrup M Khan S Schwarz K Schmidtke G. ‘Interferon-gamma inducible exchanges of 20S proteasome active site subunits: Why?’. Biochimie (2001) 83(3-4):367–72. doi: 10.1016/s0300-9084(01)01251-2 11295499

[51] Allen PM Unanue ER. ‘Antigen processing and presentation by macrophages’. Am J Anat (1984) 170(3):483–90. doi: 10.1002/aja.1001700319 6433692

[52] Akbar SM Inaba K Onji M. ‘Upregulation of MHC class II antigen on dendritic cells from hepatitis b virus transgenic mice by interferon-gamma: abrogation of immune response defect to a T-cell-dependent antigen’. Immunology (1996) 87(4):519–27. doi: 10.1046/j.1365-2567.1996.516576.x PMC1384128 8675204

[53] Rongcun Y Maes H Corsi M Dellner F Wen T Kiessling R. ‘Interferon gamma impairs the ability of monocyte-derived dendritic cells to present tumour-specific and allo-specific antigens and reduces their expression of CD1A, CD80 AND CD4’. Cytokine (1998) 10(10):747–55. doi: 10.1006/cyto.1998.0357 9811527

[54] Thelemann C Eren RO Coutaz M Brasseit J Bouzourene H Rosa M. ‘Interferon-γ induces expression of MHC class II on intestinal epithelial cells and protects mice from colitis’. PLoS One (2014) 9(1):e86844. doi: 10.1371/journal.pone.0086844 24489792 PMC3904943

[55] Xaus J Cardó M Valledor AF Soler C Lloberas J Celada A. ‘Interferon gamma induces the expression of p21waf-1 and arrests macrophage cell cycle, preventing induction of apoptosis’. Immunity (1999) 11(1):103–13. doi: 10.1016/s1074-7613(00)80085-0 10435583

[56] Todd PA Goa KL. ‘Interferon gamma-1b’. Drugs (1992) 43(1):111–22. doi: 10.2165/00003495-199243010-00008 1372855

[57] Xu J Ji Y Shogren KL Okumo SH Yaszemski MJ Maran A. ‘RNA-dependent protein kinase is required for interferon-γ-induced autophagy in MG63 osteosarcoma cells’. Gene (2021) 802:145865. doi: 10.1016/j.gene.2021.145865 34352301

[58] Kak G Raza M Tiwari B. ‘Interferon-gamma (IFN-γ): Exploring its implications in infectious diseases’. Biomolecular Concepts (2018) 9:64–79. doi: 10.1515/bmc-2018-0007 29856726

[59] Badaro R Falcoff E Badaro FS Carvalho EM Pedral-Sampaio D Barral A. ‘Treatment of visceral leishmaniasis with pentavalent antimony and interferon gamma’. N Engl J Med (1990) 322(1):16–21. doi: 10.1056/NEJM199001043220104 2104665

[60] The International Chronic Granulomatous Disease Cooperative Study Group. ‘A Controlled Trial of Interferon Gamma to Prevent Infection in Chronic Granulomatous Disease’ . N Engl J Med (1991) 324(8):509–516. doi: 10.1056/NEJM199102213240801 1846940

[61] Anon. Effects of interferon-gamma on cavitary pulmonary tuberculosis in the lungs. Available at: https://clinicaltrials.gov/ct2/show/study/NCT00201123.

[62] Anon. Use of ACTIMMUNE in patients with ADO2. Available at: https://clinicaltrials.gov/ct2/show/study/NCT02584608.

[63] Lazear HM Schoggins JW Diamond MS. ‘Shared and distinct functions of type I and type III interferons’. Immunity (2019) 50(4):907–23. doi: 10.1016/j.immuni.2019.03.025 PMC6839410 30995506

[64] Vanderheiden A Ralfs P Chirkova T Upadhyay AA Zimmerman MG Bedoya S. ‘Type I and type III interferons restrict SARS-COV-2 infection of human airway epithelial cultures’. J Virol (2020) 94(19):e00985-20. doi: 10.1128/JVI.00985-20 32699094 PMC7495371

[65] Wack A Terczyńska-Dyla E Hartmann R. Guarding the frontiers: The biology of type III interferons. Nat Immunol (2015) 16:802–9. doi: 10.1038/ni.3212 PMC7096991 26194286

[66] Morrow MP Pankhong P Laddy DJ Schoenly KA Yan J Cisper N. ‘Comparative ability of IL-12 and IL-28B to regulate treg populations and enhance adaptive cellular immunity’. Blood (2009) 113(23):5868–77. doi: 10.1182/blood-2008-11-190520 PMC2700323 19304955

[67] Ank N Iversen MB Bartholdy C Staeheli P Hartmann R Jensen UB. ‘An important role for type III interferon (IFN-lambda/IL-28) in TLR-induced antiviral activity’. J Immunol (2008) 180(4):2474–85. doi: 10.4049/jimmunol.180.4.2474 18250457

[68] Lasfar A Gogas H Zloza A Kaufman HL Kirkwood JM. ‘IFN-λ cancer immunotherapy: new kid on the block’. Immunotherapy (2016) 8(8):877–88. doi: 10.2217/imt-2015-0021 PMC5619162 27381684

[69] Lasfar A Lewis-Antes A Smirnov SV Anantha S Abushahba W Tian B. ‘Characterization of the mouse IFN-lambda ligand-receptor system: IFN-lambdas exhibit antitumor activity against B16 melanoma’. Cancer Res (2006) 66(8):4468–77. doi: 10.1158/0008-5472.CAN-05-3653 16618774

[70] Anon. A study to evaluate pegylated interferon lambda monotherapy in patients with chronic hepatitis delta virus infection (LIMT). Available at: https://clinicaltrials.gov/ct2/show/study/NCT02765802.

[71] Anon. Study of PEG-rIL-29 (or PEF-IFN lambda) in subjects with chronic hepatitis c virus infection. Available at: https://clinicaltrials.gov/ct2/show/study/NCT00565539.

[72] Anon. Dose ranging study of pegylated interferon lambda in patients with Hepatitis B and positive for Hepatitis B e Antigen (LIRA-B) – full text view. Full Text View – ClinicalTrials.gov. Available at: https://clinicaltrials.gov/ct2/show/NCT01204762.

[73] Young SN. How to increase serotonin in the human brain without drugs. J Psychiatry Neurosci (2007) 32(6):394–9. PMC2077351 18043762

[74] Baranyi A Meinitzer A Breitenecker RJ Amouzadeh-Ghadikolai O Stauber R Rothenhäusler HB. ‘Quinolinic acid responses during interferon-α-induced depressive symptomatology in patients with chronic hepatitis c infection - a novel aspect for depression and inflammatory hypothesis’. PLoS One (2015) 10(9):e0137022. doi: 10.1371/journal.pone.0137022 26368809 PMC4569409

[75] Jayawickrama GS Nematollahi A Sun G Gorrell MD Church WB. ‘Inhibition of human kynurenine aminotransferase isozymes by estrogen and its derivatives’. Sci Rep (2017) 7(1):17559. doi: 10.1038/s41598-017-17979-7 29242525 PMC5730616

[76] Wichers MC Koek GH Robaeys G Verkerk R Scharpé S Maes M. ‘IDO and interferon-alpha-induced depressive symptoms: a shift in hypothesis from tryptophan depletion to neurotoxicity’. Mol Psychiatry (2005) 10(6):538–44. doi: 10.1038/sj.mp.4001600 15494706

[77] Maes M Mihaylova I Ruyter MD Kubera M Bosmans E. ‘The immune effects of TRYCATs (tryptophan catabolites along the IDO pathway): relevance for depression - and other conditions characterized by tryptophan depletion induced by inflammation’. Neuro Endocrinol Lett (2007) 28(6):826–31. 18063923

[78] Galvão-de Almeida A Quarantini LC Sampaio AS Lyra AC Parise CL Paraná R. ‘Lack of association of indoleamine 2,3-dioxygenase polymorphisms with interferon-alpha-related depression in hepatitis c’. Brain Behavior Immun (2011) 25(7):1491–7. doi: 10.1016/j.bbi.2011.06.001 21693183

[79] Schoedon G Troppmair J Adolf G Huber C Niederwieser A. ‘Interferon-gamma enhances biosynthesis of pterins in peripheral blood mononuclear cells by induction of GTP-cyclohydrolase I activity’. J Interferon Res (1986) 6(6):697–703. doi: 10.1089/jir.1986.6.697 3106526

[80] Kitagami T Yamada K Miura H Hashimoto R Nabeshima T Ohta T. ‘Mechanism of systemically injected interferon-alpha impeding monoamine biosynthesis in rats: role of nitric oxide as a signal crossing the blood-brain barrier’. Brain Res (2003) 978(1-2):104–14. doi: 10.1016/s0006-8993(03)02776-8 12834904

[81] Zhou Y Danbolt NC. ‘Glutamate as a neurotransmitter in the healthy brain’. J Neural Transm (2014) 121(8):799–817. doi: 10.1007/s00702-014-1180-8 24578174 PMC4133642

[82] Tavares RG Tasca CI Santos CE Alves LB Porciúncula LO Emanuelli T. ‘Quinolinic acid stimulates synaptosomal glutamate release and inhibits glutamate uptake into astrocytes’. Neurochemistry Int (2002) 40(7):621–7. doi: 10.1016/s0197-0186(01)00133-4 11900857

[83] Hoyo-Becerra C Schlaak JF Hermann DM. Insights from interferon-α-related depression for the pathogenesis of depression associated with inflammation, brain, behavior, and immunity. (2014) 42:222–31. doi: 10.1016/j.bbi.2014.06.200 25066466

[84] Chiu WC Su YP Su KP Chen P-C. ‘Recurrence of depressive disorders after interferon-induced depression’. Trans Psychiatry (2017) 7:e1026. doi: 10.1038/tp.2016.274 PMC5438022 28170005

[85] Wichers MC Maes M. The role of indoleamine 2,3-dioxygenase (IDO) in the pathophysiology of interferon-alpha-induced depression. J Psychiatry Neurosci (2004) 29:11–7. PMC305266 14719046

[86] Bonaccorso S Marino V Biondi M Grimaldi F Ippoliti F Maes M. Depression induced by treatment with interferon-alpha in patients affected by hepatitis c virus. J Affect Disorders (2002) 72(3):237–41. doi: 10.1016/S0165-0327(02)00264-1 12450640

[87] Vécsei L Beal MF. ‘Influence of kynurenine treatment on open-field activity, elevated plus-maze, avoidance behaviors and seizures in rats’. Pharmacology Biochemistry Behav (1990) 37(1):71–6. doi: 10.1016/0091-3057(90)90043-h 2263669

[88] Lapin IP Mutovkina LG Ryzov IV Mirzaev S. Anxiogenic activity of quinolinic acid and kynurenine in the social interaction test in mice. J Psychopharmacol (1996) 10(3):246–9. doi: 10.1177/026988119601000312 22302953

[89] Maes M Galecki P Chang YS Berk M. ‘A review on the oxidative and nitrosative stress (O&NS) pathways in major depression and their possible contribution to the (neuro)degenerative processes in that illness’. Prog Neuropsychopharmacol Biol Psychiatry (2011) 35(3):676–92. doi: 10.1016/j.pnpbp.2010.05.004 20471444

[90] Tsao C-W Lin Y-S Cheng J-T Lin C-F Wu H-T Wu S-R. ‘interferon-alpha-induced serotonin uptake in jurkat t cells *via* mitogen-activated protein kinase and transcriptional regulation of the serotonin transporter’. J Psychopharmacol (2008) 22:753–60. doi: 10.1177/0269881107082951 18308792

[91] Hepgul N Cattaneo A Agarwal K Baraldi S Borsini A Bufalino C. ‘Transcriptomics in interferon-*α*-treated patients identifies inflammation-, neuroplasticity- and oxidative stress-related signatures as predictors and correlates of depression’. Neuropsychopharmacology (2016) 41:2502–11. doi: 10.1038/npp.2016.50 PMC4983179 27067128

[92] Wichers M Kenis G Koek G Robaeys G Nicolson NA Maes M. ‘Interferon-alpha-induced depressive symptoms are related to changes in the cytokine network but not to cortisol’. J Psychosom Res (2007) 62(2):207–14. doi: 10.1016/j.jpsychores.2006.09.007 17270579

[93] Raison CL Borisov AS Majer M Drake DF Pagnoni G Woolwine BJ. ‘Activation of central nervous system inflammatory pathways by interferon-alpha: relationship to monoamines and depression’. Biol Psychiatry (2009) 65(4):296–303. doi: 10.1016/j.biopsych.2008.08.010 18801471 PMC2655138

[94] Renault PF Hoofnagle JH Park Y Mullen KD Peters M Jones DB. ‘Psychiatric complications of long-term interferon alfa therapy’. Arch Internal Med (1987) 147(9):1577–80. doi: 10.1001/archinte.1987.00370090055011 3307672

[95] Otsubo T Miyaoka H Kamijima K Onuki M Ishii M Mitamura K. ‘Depression during interferon therapy in chronic hepatitis c patients – a prospective study’. Seishin shinkeigaku zasshi = Psychiatria neurologia Japonica (1997) 99(3):101–27. 9136611

[96] Hauser P Khosla J Aurora H Laurin J Kling MA Hill J. A prospective study of the incidence and open-label treatment of interferon-induced major depressive disorder in patients with hepatitis c. Mol Psychiatry (2002) 7(9):942–7. doi: 10.1038/sj.mp.4001119 12399946

[97] Horikawa N Yamazaki T Izumi N Uchihara M. ‘Incidence and clinical course of major depression in patients with chronic hepatitis type c undergoing interferon-alpha therapy: A prospective study’. Gen Hosp Psychiatry (2003) 25(1):34–8. doi: 10.1016/s0163-8343(02)00239-6 12583926

[98] Kraus MR Schäfer A Faller H Csef H Scheurlen M. ‘Psychiatric symptoms in patients with chronic hepatitis c receiving interferon alfa-2b therapy’. J Clin Psychiatry (2003) 64(6):708–14. doi: 10.4088/jcp.v64n0614 12823087

[99] Dieperink E Ho SB Thuras P Willenbring ML. ‘A prospective study of neuropsychiatric symptoms associated with interferon-α-2b and ribavirin therapy for patients with chronic hepatitis c’. Psychosomatics (2003) 44(2):104–12. doi: 10.1176/appi.psy.44.2.104 12618532

[100] Dieperink E Ho SB Tetrick L Thuras P Dua K Willenbring ML. ‘Suicidal ideation during interferon-α2b and ribavirin treatment of patients with chronic hepatitis c’. Gen Hosp Psychiatry (2004) 26(3):237–40. doi: 10.1016/j.genhosppsych.2004.01.003 15121353

[101] Alavi M Grebely J Matthews GV Petoumenos K Yeung B Day C. ‘Effect of pegylated interferon-α-2a treatment on mental health during recent hepatitis c virus infection’. J Gastroenterol Hepatol (2012) 27(5):957–65. doi: 10.1111/j.1440-1746.2011.07035.x PMC3331928 22142332

[102] Pavol MA Meyers CA Rexer JL Valentine AD Mattis PJ Talpaz M. Pattern of neurobehavioral deficits associated with interferon alfa therapy for leukemia. Neurology. (1995) 45(5):947–50. doi: 10.1212/wnl.45.5.947 7746412

[103] Caraceni A Gangeri L Martini C Belli F Brunelli C Baldini M. ‘Neurotoxicity of interferon-alpha in melanoma therapy: results from a randomized controlled trial’. Cancer (1998) 83(3):482–9. doi: 10.1002/(sici)1097-0142(19980801)83:3<482::aid-cncr17>3.0.co;2-s 9690541

[104] Greenberg DB Jonasch E Gadd MA Ryan BF Everett JR Sober AJ. ‘Adjuvant therapy of melanoma with interferon-alpha-2b is associated with mania and bipolar syndromes’. Cancer (2000) 89(2):356–62. 10918166

[105] Sheng JA Bales NJ Myers SA Bautista AI Roueinfar M Hale TM. ‘The hypothalamic-Pituitary-Adrenal axis: Development, programming actions of hormones, and maternal-fetal interactions’. Front Behav Neurosci (2021) 14:601939. doi: 10.3389/fnbeh.2020.601939 33519393 PMC7838595

[106] Wyrwoll CS Holmes MC Seckl JR. ‘11β-hydroxysteroid dehydrogenases and the brain: From zero to hero, a decade of progress’. Front Neuroendocrinol (2011) 32(3):265–86. doi: 10.1016/j.yfrne.2010.12.001 PMC3149101 21144857

[107] Mikulska J Juszczyk G Gawrońska-Grzywacz M Herbet M. ‘HPA axis in the pathomechanism of depression and schizophrenia: New therapeutic strategies based on its participation’. Brain Sci (2021) 11(10):1298. doi: 10.3390/brainsci11101298 34679364 PMC8533829

[108] Thau L Gandhi J Sharma S. Physiology, cortisol. Treasure Island, Florida (FL): StatPearls Publishing (2022). Available at: https://www.ncbi.nlm.nih.gov/books/NBK538239/. 30855827

[109] Hanson RW Owen OE. ‘Gluconeogenesis’. In: Lennarz WJ Lane MD, editors. Encyclopedia of biological chemistry (Second edition). Waltham: Academic Press (2013). p. 381–6. doi: 10.1016/B978-0-12-378630-2.00040-2

[110] Marin MF Lord C Andrews J Juster RP Sindi S Arsenault-Lapierre G. ‘Chronic stress, cognitive functioning and mental health’. Neurobiol Learn Memory (2011) 96(4):583–95. doi: 10.1016/j.nlm.2011.02.016 21376129

[111] Hill MN Tasker JG. ‘Endocannabinoid signaling, glucocorticoid-mediated negative feedback, and regulation of the hypothalamic-pituitary-adrenal axis’. Neuroscience (2012) 204:5–16. doi: 10.1016/j.neuroscience.2011.12.030 22214537 PMC3288468

[112] Joseph DN Whirledge S. ‘Stress and the HPA axis: Balancing homeostasis and fertility’. Int J Mol Sci (2017) 18(10):2224. doi: 10.3390/ijms18102224 29064426 PMC5666903

[113] Bielajew C Konkle AT Merali Z. ‘The effects of chronic mild stress on male sprague-dawley and long Evans rats: I. biochemical and physiological analyses’. Behav Brain Res (2002) 136(2):583–92. doi: 10.1016/s0166-4328(02)00222-x 12429420

[114] Bertollo AG Grolli RE Plissari ME Ignácio ZM Gasparin VA Quevedo J. ‘Stress and serum cortisol levels in major depressive disorder: a cross-sectional study’. AIMS Neurosci (2020) 7(4):459–69. doi: 10.3934/Neuroscience.2020028 PMC7701370 33263081

[115] Magariños AM Orchinik M McEwen BS. ‘Morphological changes in the hippocampal CA3 region induced by non-invasive glucocorticoid administration: a paradox’. Brain Res (1998) 809(2):314–8. doi: 10.1016/s0006-8993(98)00882-8 9853126

[116] Sterner EY Kalynchuk LE. ‘Behavioral and neurobiological consequences of prolonged glucocorticoid exposure in rats: relevance to depression’. Prog Neuropsychopharmacol Biol Psychiatry (2010) 34(5):777–90. doi: 10.1016/j.pnpbp.2010.03.005 20226827

[117] Murialdo G Barreca A Nobili F Rollero A Timossi G Gianelli MV. ‘Relationships between cortisol, dehydroepiandrosterone sulphate and insulin-like growth factor-I system in dementia’. J Endocrinological Invest (2001) 24(3):139–46. doi: 10.1007/BF03343833 11314741

[118] Miranda M Morici JF Zanoni MB Bekinschtein P. ‘Brain-derived neurotrophic factor: A key molecule for memory in the healthy and the pathological brain’. Front Cell Neurosci (2019) 13:363. doi: 10.3389/fncel.2019.00363 31440144 PMC6692714

[119] Yue Y Yuan Y Hou Z Jiang W Bai F Zhang Z. ‘Abnormal functional connectivity of amygdala in late-onset depression was associated with cognitive deficits’. PLoS One (2013) 8(9):e75058. doi: 10.1371/journal.pone.0075058 24040385 PMC3769296

